# Evolutionary mechanism leading to the multi-*cagA* genotype in *Helicobacter pylori*

**DOI:** 10.1038/s41598-019-47240-2

**Published:** 2019-08-01

**Authors:** Hanfu Su, Kavinda Tissera, Sungil Jang, Yun Hui Choi, Aeryun Kim, Yong-Joon Cho, Meiling Li, Niluka Gunawardhana, D. Scott Merrell, Linhu Ge, Jeong-Heon Cha

**Affiliations:** 1 Key Laboratory of Oral Medicine, Guangzhou Institute of Oral Disease, Affiliated Stomatology Hospital of Guangzhou Medical University, Guangzhou, China; 2 Department of Oral Biology, Oral Science Research Center, Department of Applied Life Science, The Graduate School, BK21 Plus Project, Yonsei University College of Dentistry, Seoul, Republic of Korea; 3 Department of Oral Biochemistry, Chonbuk National University School of Dentistry, Jeonju, Republic of Korea; 4 Division of Polar Life Science, Korea Polar Research Institute, Incheon, Republic of Korea; 5 Department of Basic Sciences, Faculty of Dental Sciences, University of Peradeniya, Peradeniya, Sri Lanka; 6 Department of Microbiology and Immunology, Uniformed Services University of the Health Sciences, Bethesda, Maryland USA

**Keywords:** Bacterial genetics, Pathogens, Bacterial pathogenesis, Bacterial genomics, Prokaryote

## Abstract

Infection with CagA+ *Helicobacter pylori* strains is linked to an increased risk for gastric diseases, including gastric cancer. Recent evidence indicates that dynamic expansion and contraction of *cagA* copy number may serve as a novel mechanism to enhance disease development. Herein, comparative genomic analysis divided hpEurope into two groups: hpEurope/type-A and type-B. Only hpEurope/type-B displayed the multi-*cagA* genotype. Further analysis showed that *cag*PAI appears to have been independently introduced into two different *H*. *pylori* types, termed pre-type-A and pre-type-B, which consequently evolved to *cag*PAI type-A and type-B, respectively; importantly, all multi-*cagA* genotype strains displayed *cag*PAI type-B. Two direct *cagA*-flanking repeats of a genetic element termed CHA-ud were essential for the multi-*cagA* genotype in strain PMSS1 (hpEurope/type-B and *cag*PAI type-B). Furthermore, introduction of this genetic element into strain G27 (hpEurope/type-A and *cag*PAI type-A) was sufficient to generate the multi-*cagA* genotype. The critical steps in the evolution of the multi-*cagA* genotype involved creation of CHA-ud at *cagA* upstream in *cag*PAI type-B strains followed by its duplication to *cagA* downstream. *En masse*, elucidation of the mechanism by which *H*. *pylori* evolved to carry multiple copies of *cagA* helps to provide a better understanding of how this ancient pathogen interacts with its host.

## Introduction

*Helicobacter pylori* is a Gram-negative pathogen that inhabits the gastric mucosa of a significant portion of the human population and causes gastric diseases with severities that range from gastritis to gastric adenocarcinoma and mucosa-associated lymphoid tissue (MALT) lymphoma^1,2^. Once colonized, *H*. *pylori* infection chronically persists without treatment. Gastric cancer occurs at a prevalence of 1–2% in *H*. *pylori* infected subjects^3^. Given the association of *H*. *pylori* with gastric cancer and the fact that more than half of the world’s population is infected with this microbe, gastric carcinoma is the third leading cause of cancer-related death worldwide^4^. Accordingly, *H*. *pylori* is classified as a class I carcinogen by the World Health Organization^5^.

Modern humans were infected by *H*. *pylori* long before their first successful migration out of Africa 60 thousand years ago^6,7^. Since then, *H*. *pylori* has coevolved with human beings such that strains from different geographic regions have differentiated into *H*. *pylori* populations that are generally tightly associated with the human populations found in those areas. As a result, genetic differences seen among various *H*. *pylori* strains elicits interests in terms of evolution and virulence. Indeed, Multi Locus Sequence Typing (MLST) has been used to classify *H*. *pylori* into seven large population types: hpAfrica1, hpAfrica2, hpNEAfrica, hpEurope, hpEastAsia, hpAsia2 and hpSahul^6,7^. While the super-lineage hpAfrica2 was derived from the most ancestral *H*. *pylori* strains that migrated toward South Africa, the other populations descended from other super-lineages that migrated toward east and west Africa, and subsequently out of Africa. Migration of *H*. *pylori* with human beings out of Africa led to the establishment of two genetically distant populations, hpEurope and hpEastAsia, in which the strains were mainly isolated from Western and East Asian countries, respectively. In addition, before high-throughput sequencing methods became popular, prior studies considered gene arrangement in the *H*. *pylori* chromosome; McGee *et al*. speculated that strains 26695 and 43504 have different genome organizations, and Blomstergren *et al*. reported a strain that carried a large insertion in the *cag* Pathogenicity Island (*cag*PAI) region^8,9^. With the advent of modern sequencing technology and comparative genome analysis, Farnbacher *et al*. identified chromosome inversion pattern of strain B8, and further showed that B8 has a different gene arrangement in the *cag*PAI region as compared to other *H*. *pylori* strains^10^. Thus, due to the ability of *H*. *pylori* to rearrange its genome, different *H*. *pylori cag*PAI arrangements have been appreciated.

Among the various virulence factors of *H*. *pylori*, the oncoprotein cytotoxin-associated gene A (CagA) is thought to play a central role as a scaffolding protein in the development of gastric cancer^11^. The CagA effector is injected into host cells by a syringe-like structure termed the type IV secretion system (T4SS). Both CagA and the T4SS are encoded on a 40-kb DNA segment known as the *cag*PAI, which generally consists of 27 genes^12^. The *cag*PAI region is thought to have been introduced into the *H*. *pylori* genome via horizontal transfer from an unknown source. Once translocated to host cells, CagA undergoes phosphorylation by host cell kinases at a conserved tyrosine residue found within the EPIYA (Glu-Pro-Ile-Tyr-Ala) motif. CagA is known to interact with a wide variety of host cell signaling molecules and to perturb normal host cell signaling^13,14^. For example, phosphorylated CagA binds to a SH2-domain-containing protein tyrosine phosphatase (SHP2), and thus deregulates the phosphatase activity of SHP2^15^. This in turn leads to hyperstimulation of Ras-Erk signaling.

In a recent study, we identified *H*. *pylori* strains that harbor multiple copies of the *cagA* gene, and showed that dynamic modulation of *cagA* copy number may impact development of gastric disease^16^. The multi-*cagA* genotype was only found in a subset of strains those were part of a collection of clinical isolates from the United States; this genotype was not seen in a large clinical collection from South Korea. Given this, we reasoned that the distribution of the multi-*cagA* genotype in the Western *H*. *pylori* population required further investigation. Furthermore, the mechanism by which *H*. *pylori* acquired the ability to modulate *cagA* copy number during coevolution with the human host remains to be elucidated. Thus herein, we performed PacBio whole genome sequencing of all of the clinical multi-*cagA* isolates we identified. The sequences were used as the foundation for comparative genomic analyses of a larger number of *H*. *pylori* strains that represent isolates derived from different worldwide geographic origins. We found that the multi-*cagA* genotype was only seen in a subset of hpEurope, termed hpEurope/type-B. Furthermore, we defined an essential *cagA* flanking genetic element that is required for generation of the multi-*cagA* genotype. Finally, we propose an evolutionary mechanism by which *H*. *pylori* developed the multi-*cagA* genotype. Overall, these results aid our understanding of *H*. *pylori* pathogenesis and its coevolution with the human host.

## Results

### General genomic features of all multi-*cagA H*. *pylori* clinical isolates

To begin to understand the process by which *H*. *pylori* developed the multi-*cagA* genotype, we sought to define the complete genome sequence of all identified *H*. *pylori* clinical isolates shown to carry multiple copies of *cagA*. Thus, we performed whole genome PacBio sequencing of six *H*. *pylori* strains containing the multi-*cagA* genotype (B125A, B128, B130A, B140, B147 and 7.13) along with two *H*. *pylori* strains containing a single-*cagA* (B136A and J182) (Table 1). Sequencing of each of the 8 strains resulted in a single circularized chromosome. Overall, these chromosomes showed a typical *H*. *pylori* genome GC content of 38.71–38.90% with a chromosomal length that ranged from approximately 1,646 kbp to 1,727 kbp. Of note, the genome sequences of three additional multi-*cagA* strains, PMSS1 (along with its mouse passaged derivative SS1) and J166, were released by other research groups^17,18^; our subsequent analyses included these whole genome sequences.

**Table 1:** General features for *H*. *pylori* genome sequences of nine multi-*cagA* genotype strains and two single-*cagA* genotype strains (*).

Strains	Genome size (bp)	GC %	CDS (coding)	CDS (total)	Reference
PMSS1	1,618,480	38.99	1,438	1,543	^17^
SS1	1,619,098	38.99	1,432	1,540	^17^
J166	1,650,561	38.90	1,459	1,563	^18^
7.13	1,676,743	38.79	1,492	1,598	this study
B125A	1,656,704	38.90	1,459	1,566	this study
B128	1,675,360	38.79	1,490	1,600	this study
B130A	1,646,042	38.87	1,441	1,566	this study
B140	1,726,836	38.71	1,521	1,653	this study
B147	1,631,093	38.90	1,436	1,554	this study
B136A*	1,663,520	38.90	1,475	1,601	this study
J182*	1,648,077	38.90	1,499	1,584	this study

### All multi-*cagAH*. *pylori* strains belong to hpEurope

**Figure 1:**
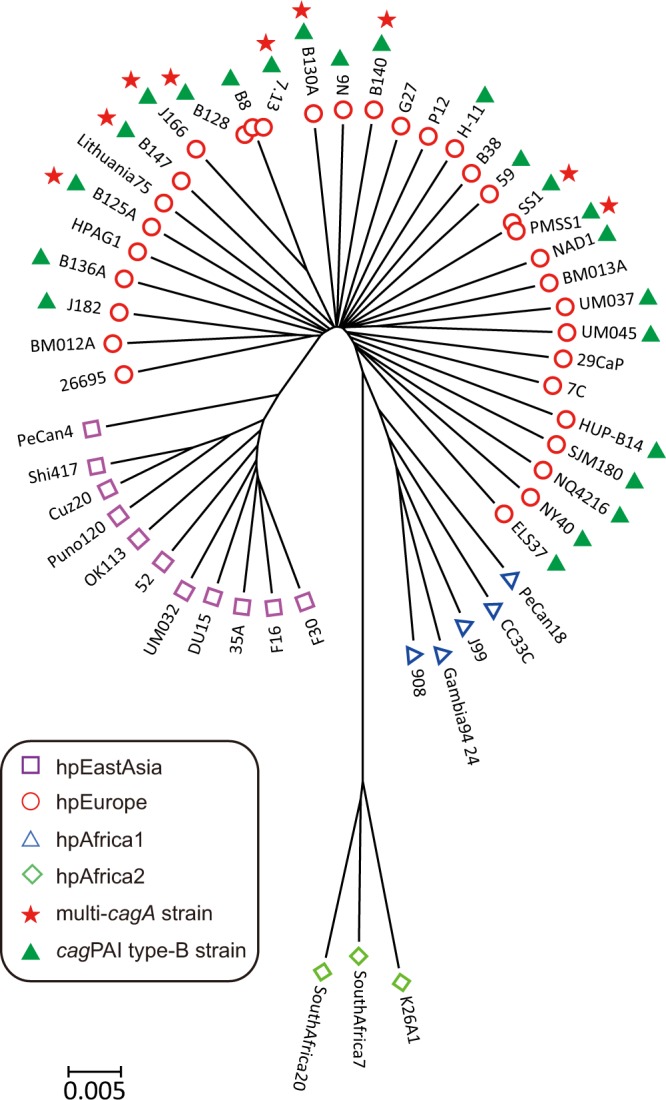
Neighbor-joining tree of the 52 *H*. *pylori* strains based on core genome pairwise distance. The Neighbor-joining tree was calculated from concatenated nucleic acid sequences of 927 orthologous genes (length 855,682 bp). Each *H*. *pylori* population and strains within it are indicated. The 9 multi-*cagA* genotype *H*. *pylori* strains grouped with hpEurope strains. The *cag*PAI type-B strains are defined in a latter section.

Our prior analysis showed that the *H*. *pylori* multi-*cagA* genotype was found in American isolates but not in South Korean isolates^16^; thus, suggesting that the multi-*cagA* genotype is geographically-associated. To further categorize the multi-*cagA* strains, we performed phylogenetic analysis of the nine strains displaying this genotype (including the SS1 strain), with an additional 43 other *H*. *pylori* strains for which there were whole genome sequences available (Supplemental Table S1); this was accomplished via analysis of core genome pairwise distance (Fig. 1). The Neighbor-joining tree clearly classified the 52 *H*. *pylori* strains into hpAfrica1, hpAfrica2, hpEurope and hpEastAsia as previously described^6,7^. Of note, all strains carrying multiple copies of *cagA* belonged to hpEurope; this finding further suggests that the multi-*cagA* strains underwent a unique region-associated evolutionary history.

### The multi-*cagA* genotype is associated with hpEurope/type-B

**Figure 2:**
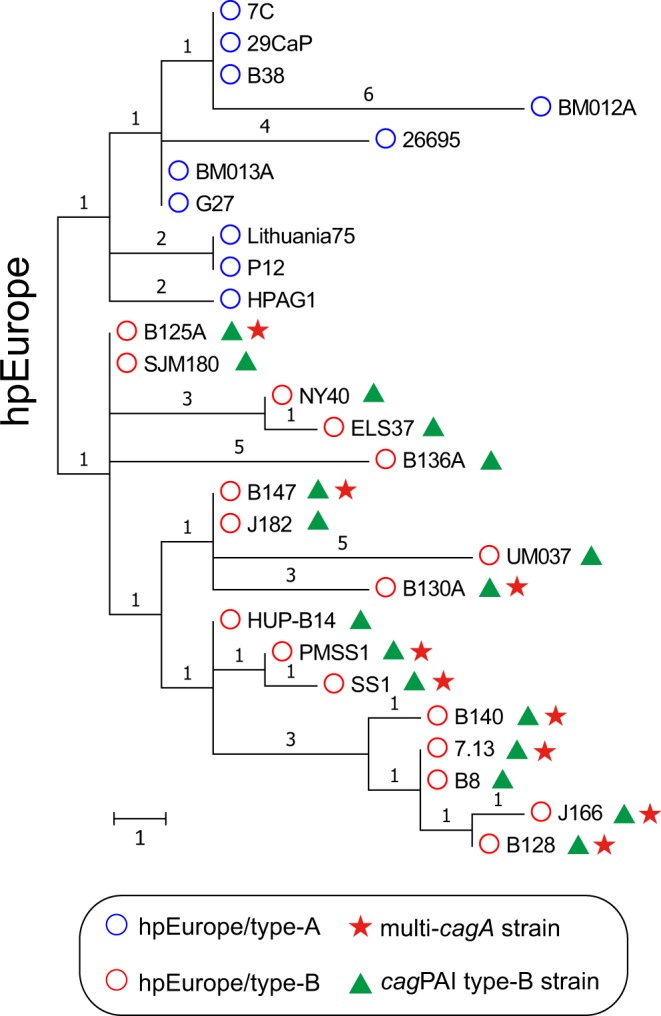
Phylogenetic relationship of the 27 hpEurope strains for which a complete genome was available. A total of 80 local colinear blocks from each of the 27 genomes were converted to a signed permutation. The signed permutation was further used to infer their phylogenetic relationship based on chromosomal reversals with a unichromosomal circular model. The tree was mid-rooted as shown in the resulting dendrogram. The two clades into which hpEurope segregated were named hpEurope/type-A and type-B and are indicated by colors as described in the figure inset. The multi-*cagA* genotype strains all segregated into hpEurope/type-B. Scale bar indicates length of one rearrangement. The number of rearrangement is denoted on each branch.

Genome rearrangement is an important factor that shapes genome structure and likely impacts gene expression^19–21^. This is especially true in *H*. *pylori* where many virulence gene polymorphisms are suggested to be the result of genome rearrangement^22^. Genomic rearrangement, as inferred by chromosomal reversal, is also commonly used to study bacterial phylogeny^23–26^. Thus, we analyzed the phylogenetic relationship inferred by chromosomal reversal distance using 46 of the 52 *H*. *pylori* strains in Fig. 1 (Supplemental Fig. S1); genome sequences of 6 *H*. *pylori* strains (N6, H-11, 59, NAD1, UM045 and NQ4216) were not completed and were excluded for this analysis. Importantly, most of the multi-*cagA H*. *pylori* strains were clustered together while the NY40 and ELS37 hpEurope stains clustered close to hpAfrica1 (Supplemental Fig. S1). Since all of the multi-*cagA H*. *pylori* strains belong to hpEurope, we further analyzed the phylogenetic relationship inferred by chromosomal reversal distance using just the 27 *H*. *pylori* strains that were assigned to hpEurope by core genome pairwise distance (Fig. 2); the whole-genome sequences of these 27 hpEurope strains were assembled as single circular contigs and thus provided full information on the genome structure. Analysis of these strains revealed that they further divided into two types, which we named hpEurope/type-A and type-B. Strikingly, the 9 multi-*cagA* strains were all classified as hpEurope/type-B along with SJM180, NY40, ELS37, B136A, J182, UM037, HUP-B14 and B8; the remaining 10 strains grouped into hpEurope/type-A.

### The multi-*cagA* genotype co-occurs with a specific *cag*PAI rearrangement called type-B

**Figure 3:**
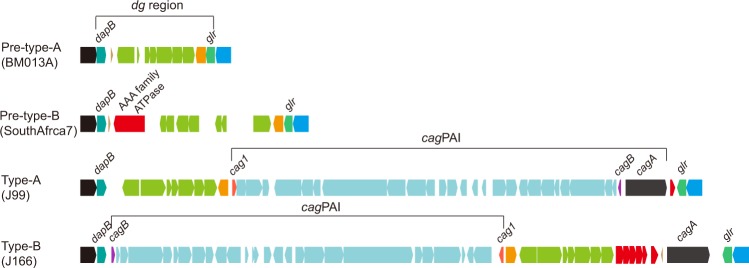
Four types of gene arrangements within the chromosomal region delimited by *dapB* and *glr* (*dg* region). Representatives of each type of gene arrangements are shown and strain names are indicated in parentheses. Genes are drawn with filled arrows and DNA homology across the strain types are indicated by the same color. The *dg* region and *cag*PAI are indicated.

Our prior study showed that *H*. *pylori* PMSS1 dynamically alters *cagA* gene copy number. Furthermore, we previously suggested that the process by which this occurs likely involves homologous recombination at the repeated *cagA* homologous area (CHA)-ud sequences those flank *cagA*; the presence, pattern and number of copies of the CHA-ud sequence varies among different strains^16^. Since the multi-*cagA* genotype appears to play an important role in the development of gastric disease^16^, we sought to define the evolutionary mechanism behind development of the multi-*cagA* genotype. In *cag*PAI positive *H*. *pylori* strains, *cag*PAI is located within a chromosomal region delimited by the *dapB* and *glr* genes, designated as the *dg* region (Fig. 3). We first compared the *dg* regions in a total of 243 *H*. *pylori* strains (Supplemental Table S2). Among these strains, the overall gene arrangements of the *dg* region grouped into four types; pre-type-A, pre-type-B, type-A and type-B, which were represented by strain BM013A, SouthAfrica7, J99 and J166, respectively (Fig. 3). Most of the analyzed strains were *cag*PAI positive (73%, 178 out of 243) while about 27% (65 out of 243) were *cag*PAI negative (Table 2). Among the *cag*PAI negative strains, the majority of the strains (88%, 57 out of 65) were pre-type-A and the remaining (12%, 8 out of 65) were pre-type-B. Among the *cag*PAI positive strains, the majority (87%, 155 out of 178) were type-A and the remaining (13%, 23 out of 178) were type-B. Specifically, besides the 9 multi-*cagA* strains, which were type-B, the other 14 type-B strains were also shown to be part of hpEurope (Fig. 1).

**Table 2:** Four types of *dg* region in the 243 *H*. *pylori* strains.

Four types of *dg* region			
*cag*PAI negative (27%, 65/243)		*cag*PAI positive (73%, 178/243)	
pre-type-A	pre-type-B	type-A	type-B
57 (88%)	8 (12%)	155 (87%)	23 (13%)

The pre-type-A strains (see BM013A in Fig. 3) carried a cluster of approximately nine genes within the *dg* region; interestingly, in the pre-type-B strains (see SouthAfrica7 in Fig. 3) seven genes of this cluster were inverted and, more importantly, an additional putative AAA family ATPase was inserted near the front of this cluster. The type-A strains (see J99 in Fig. 3) harbored an intact *cag*PAI upstream of the *glr* gene, while the type-B strains (see J166 in Fig. 3) harbored two separated *cag*PAI regions; the majority of the *cag*PAI (28 genes) was inverted downstream of the *dapB* gene and the *cagA* gene was separately located upstream of the *glr* gene.

**Figure 4:**
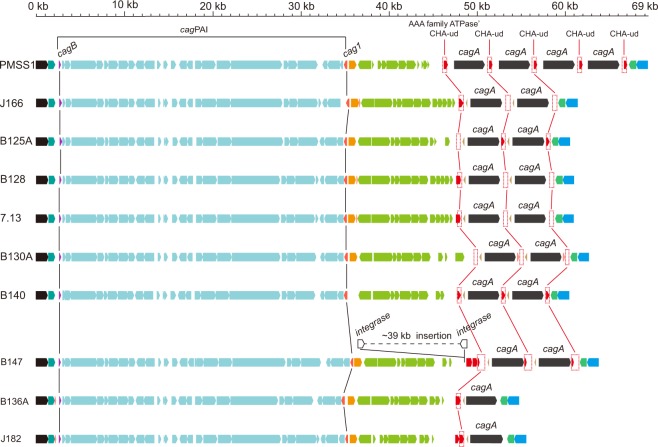
Gene arrangement within the *dg* region of strains with the multi-*cagA* genotype. All the strains carrying multiple copies of *cagA* harbored the type-B *dg*. Genes are drawn with filled arrows and DNA homology across the strain types is indicated by the same color. For comparison, the *cagA* copy number in strain J166, B125A, B128, 7.13, B130A, B140 and B147 is drawn as two copies. The first CHA-ud sequence upstream of *cagA* appears to have arisen from part of a AAA family ATPase gene. In strain B147, a ~39 kb insertion existed between CV730_03420 and CV730_03570. This insertion was delimited by two integrase genes indicated by empty arrows. The *cag*PAI region and CHA-ud are indicated.

Using the PacBio genomic sequencing and targeted Sanger dideoxy sequencing results, the *dg* regions of all of the multi-*cagA* strains were compared in Fig. 4. Notably, all of the multi-*cagA* strains belonged to *cag*PAI type-B. Furthermore, CHA-ud sequences were always found flanking *cagA* in these strains. Since all the multi-*cagA* strains harbored *cag*PAI type-B, we further asked a reciprocal question; do all of the type-B strains contain multiple copies of *cagA*? To assess this, we screened 234 clinical isolates obtained from South Korea and 80 clinical isolates obtained from the United States. We designed two sets of PCR primers (dapB-F/glnA-R and dapB-F/cagD-R) to enable us to identify the *cag*PAI type according to the position and orientation of the *cag*PAI in the *dg* region (Supplemental Fig. S2, Supplemental Table S3). As a result, we identified two more type-B strains (B136A and J182) from the United States (Table 1, Fig. 4). However, the multi-*cagA* genotype was not detected in these two strains. For further detailed analysis, we also performed whole genome sequencing of these two strains using PacBio technology (Table 1). Comparison of the *dg* region revealed that even though these two strains harbor a *cag*PAI type-B, only one CHA-ud sequence was present upstream of *cagA* (Fig. 4). Consistent with our prior results, these two strains, as well as some other *cag*PAI type-B strains (SJM180, NY40, ELS37, UM037, HUP-B14 and B8) also belonged to hpEurope/type-B. Therefore, all hpEurope/type-B strains analyzed in this study were *cag*PAI type-B strains (Fig. 2).

**Figure 5:**
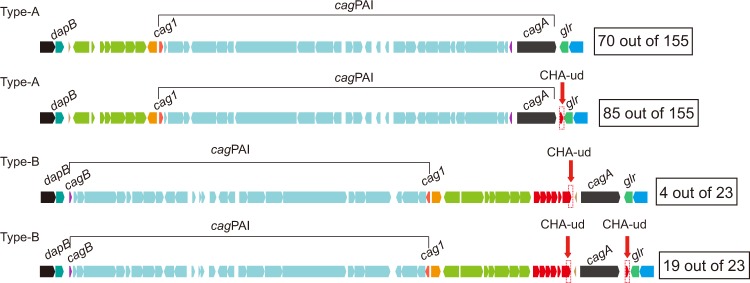
CHA-ud sequence status in *cagA*-positive strains. The representative CHA-ud status within the *dg* regions of the 178 *cagA*-positive strains is shown. Genes are drawn with filled arrows and DNA homology across the strain types is indicated by the same color. The CHA-ud position is indicated. The number of strains showing each type of CHA-ud status is indicated in the box.

To further characterize the number and location of the CHA-ud sequence, we searched for the CHA-ud sequence among the 178 *cag*PAI positive strains (Fig. 5). As a result, we discovered a relationship between CHA-ud and the *cag*PAI type. The CHA-ud only occurred within the *dg* region. Strains with *cag*PAI type-A possessed 0 or 1 CHA-ud; in these CHA-ud positive strains, CHA-ud always only appeared downstream of *cagA*. In comparison, strains with *cag*PAI type-B possessed 1 or 2 CHA-ud. In strains with one CHA-ud, the sequence always appeared upstream of *cagA*. Conversely, in strains with two CHA-ud, the sequences always flanked *cagA*. Given our prior supposition that *cagA*-flanking CHA-ud sequences were required for the homologous recombination that likely generates the multi-*cagA* genotype^16^, these data suggest that only *cag*PAI type-B strains containing two CHA-ud sequences have the potential to possess multiple *cagA* genes. Importantly, regardless of the *cagA* copy number, all of the 23 *cag*PAI type-B strains were part of the hpEurope clade (Figs 1 and 5). Specifically, the 17 strains with complete genome sequences were hpEurope/type-B (Fig. 2). Due to the incompleteness of the genome sequences of the remaining 6 *cag*PAI type-B strains, their groups  in the hpEurope/type-A or type-B remain unclear. These data suggest that hpEurope/type-B strains underwent chromosomal rearrangements that resulted in the potential for the multi-*cagA* genotype.

### Genetic element required for generation of the multi-*cagA* genotype

**Figure 6:**
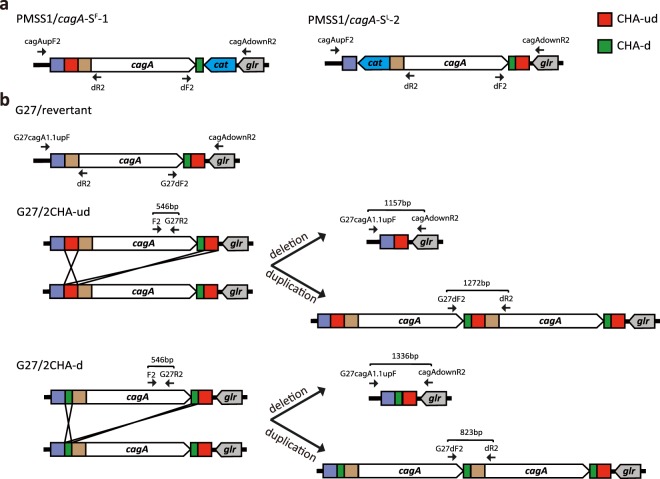
Illustration of *cagA* copy number in PMSS1 and G27 isogenic mutant strains. The *cagA* gene copy number status in PMSS1 and G27 isogenic mutant strains were detected using colony PCR; and the *cagA* regions were confirmed by Sanger sequencing as described in Methods. Both PMSS1 isogenic mutant strains and the G27/revertant showed no changes in the *cagA* region; conversely, G27/2CHA-ud and G27/2CHA-d were able to delete and duplicate the *cagA* gene. Primer annealing sites for both colony PCR and sequencing as well as length of PCR amplicons are indicated. Recombination events in the *cagA* region are shown.

Despite the previous work with *H*. *pylori* PMSS1 that suggested that two CHA-ud repeats are important for generation of the multiple *cagA* genotype and for deletion of *cagA*^16^, the mechanism behind the *cagA* duplication and deletion had not been explored experimentally. Therefore, we asked whether two direct CHA-ud repeats that flank *cagA* were essential for generation of the multiple *cagA* genotype using PMSS1 isogenic mutant strains, PMSS1/*cagA*-S^F^-1 and -S^L^-2 strains^16^. The PMSS1/*cagA*-S^F^-1 mutant strain contains a single CHA-ud upstream of *cagA* and the PMSS1/*cagA*-S^L^-2 mutant strain contains a single CHA-ud downstream of *cagA* (Fig. 6a). Using two previously described PCR strategies^16^, we screened 200 individual colonies derived from each PMSS1/*cagA*-S^F^-1 and -S^L^-2 strains for *cagA* duplication as well as deletion; we reasoned that recombination between CHA-ud elements could result in both possible genotypes. Representative PCR data were shown in Supplemental Fig. S3A. No duplication or deletion events of *cagA* were detected, further suggesting that two direct CHA-ud repeats that flank *cagA* are essential for evolution of the multiple *cagA* genotype.

Given this, we next asked if introduction of two direct 449-bp CHA-ud repeats flanking *cagA* would be sufficient to generate the multi-*cagA* genotype in G27 *H*. *pylori*. G27 possesses *cag*PAI type-A with only one CHA-ud downstream of *cagA* (Fig. 3, Type-A). A markerless gene editing method using a *kan-sacB* cassette (*ks*c) was utilized to insert one additional 449-bp CHA-ud or one extra 179-bp sequence (designated as CHA-d) upstream of *cagA* (Supplemental Fig. S4). Four constructs (A-D) were designed; construct A was used to add *ksc* upstream of *cagA*, resulting in G27*/ksc*; construct B was used to remove *ks*c from G27*/ksc*, resulting in G27/revertant; construct C was used to replace *ksc* with CHA-ud, resulting in G27/2CHA-ud, and construct D was used to replace *ksc* with CHA-d, resulting in G27/2CHA-d. The resulting G27/2CHA-ud and G27/2CHA-d strains contained two direct repeats of CHA-ud and CHA-d flanking *cagA*, respectively. Two different colony PCR strategies using primer sets of G27dF2/dR2 and G27cagA1.1upF/cagAdownR2, were used to screen for *cagA* duplication and deletion in the G27/revertant, G27/2CHA-ud and G27/2CHA-d (Fig. 6b); representative PCR data were shown in Supplemental Fig. S3B. While the G27/2CHA-ud and G27/2CHA-d strains were both able to undergo recombination that resulted in *cagA* duplication and deletion, the G27/revertant did not show any recombination at this region. The *cagA* duplication and deletion seen in the G27/2CHA-ud and G27/2CHA-d strains were confirmed by Sanger dideoxy sequencing. Thus, introduction of two direct CHA-ud repeats or CHA-d repeats such that they flank *cagA* was sufficient to generate the multi-*cagA* genotype in G27 *H*. *pylori*.

Levels of CagA expression in five representative *H*. *pylori* single colony isolates of the G27/revertant, G27/2CHA-ud and G27/2CHA-d were measured by Western blot (Supplemental Fig. S5). As expected, the G27/revertant strains showed similar CagA expression levels to that of wild-type G27. Interestingly, CagA expression levels in the G27/2CHA-ud and G27/2CHA-d strains were also similar to that of the G27 wild-type and G27/revertant strains. In addition, wild-type G27, G27/revertant, G27/2CHA-ud and G27/CHA-d strains were all observed to translocate and phosphorylate CagA in the human gastric adenocarcinoma (AGS) cell line (Supplemental Fig. S5).

### Evolutionary mechanism leading to the multi-*cagA* genotype

**Figure 7:**
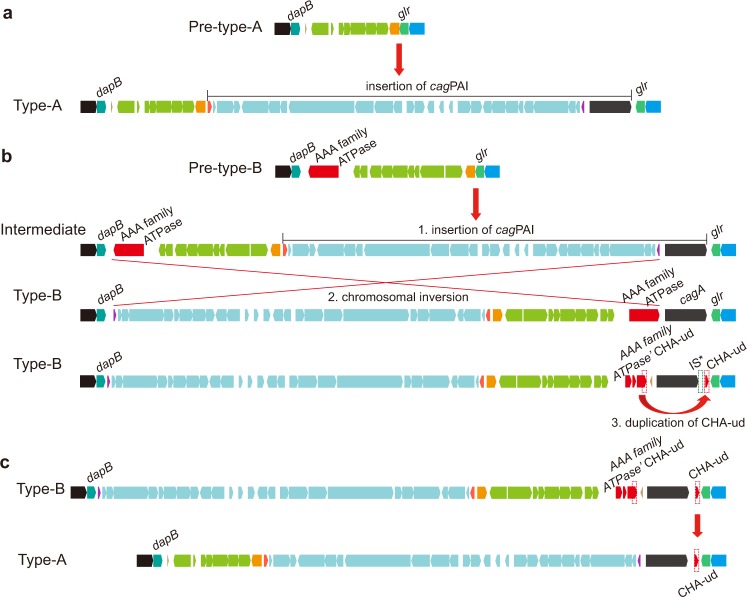
Model for the evolutionary mechanism utilized to create the multi-*cagA* genotype. The *dg* region is shown. Genes are drawn with filled arrows and DNA homology across the strain types is indicated by the same color. (**a**) Formation of the type-A *dg* by insertion of the *cag*PAI into pre-typeA *dg*. (**b**) Formation of the multi-*cagA* genotype in type-B *dg* strains by three steps. IS* indicates an insertion sequence with a transposase in PMSS1. (**c**) Homologous recombination introduced CHA-ud into type-A strains.

The above data allowed us to develop a model by which *H*. *pylori* could evolve to possess multiple copies of the *cagA* gene (Fig. 7). As mentioned previously, the *cag*PAI in *H*. *pylori* is believed to have been obtained from an unknown source via horizontal recombination^27^. Our phylogenetic analysis based on chromosomal reversal indicated that the strains in hpEurope present at least two distinct groups in terms of chromosomal structure. We also observed that among the total 243 strains analyzed, the ratio (6.3) of pre-type-A to pre-type-B strains was comparable to the ratio (6.7) of type-A to type-B strains. Therefore, we assume that prior to acquisition of the *cag*PAI, *H*. *pylori* evolved into two ancestral branches that are represented by pre-type-A and pre-type-B. These two ancestral branches then obtained *cag*PAI and became type-A and type-B, respectively. As represented by the majority of the *cag*PAI positive strains (63.8%, 155/243), one-step insertion of an intact *cag*PAI immediately upstream of *glr* in pre-type-A resulted in the formation of type-A (Fig. 7a). However, two steps were necessary for the evolution of type-B from pre-type-B. First, an intact *cag*PAI inserted upstream of *glr*, which is the same locus utilized in type-A. Second, the region delimited by the gene encoding a putative AAA family ATPase and *cagB* underwent inversion, resulting in the type-B strains (Fig. 7b). This inversion resulted in translocation of the ATPase gene to the region of upstream of *cagA*. Indeed, CHA-ud is in fact the 3′ terminal portion of this ATPase gene; thus, this inversion would explain the fact that all of the type-B strains carried CHA-ud upstream of *cagA*. Finally, a latter event duplicated CHA-ud such that it also appeared downstream of *cagA*; this may have involved activity of an insertion sequence (IS) since an IS is found in the *cagA* homologous area. Together, the two direct CHA-ud sequences then facilitated alteration of *cagA* copy number by homologous recombination.

In comparison, we note that in type-A strains, the CHA-ud found downstream of *cagA* likely had a different evolution history. This assertion is because the intact putative AAA family ATPase gene that appears to be the origin of CHA-ud, was neither found in pre-type-A nor in type-A genomes. Thus, intra-genomic recombination could not be the source of CHA-ud. Instead, since regions of *cagA* downstream are highly homologous in type-A and type-B strains, the CHA-ud likely came from inter-genomic recombination with a type-B strain (Fig. 7c). This fact may also explain why CHA-ud never appeared upstream of *cagA* in type-A strains; the upstream regions of *cagA* in type-A and type-B strains show little homology. The notion that the CHA-ud in type-A strains came from transformation and horizontal recombination of genetic material from type-B strains is supported by the fact that recombination among strains during mixed infections with multiple *H*. *pylori* strains are common^28–30^.

## Discussion

*H*. *pylori* strain PMSS1 was previously shown to represent a heterogeneous population in terms of *cagA* copy number, harboring zero to four copies of *cagA*^16^. Moreover, *cagA* copy number was dynamic as it was able to expand and contract within individual isolates. A higher *cagA* copy number was correlated with a strain’s virulence potential; strains with more copies of *cagA* produced more CagA and induced higher cell elongation and more IL-8 expression. In addition, the multi-*cagA* strains were only found among American clinical isolates but not in Korean clinical isolates.

Herein we utilized phylogenetic analysis based on core genome pairwise distance to confirm that the multi-*cagA* strains genetically belong to hpEurope (Fig. 1). For phylogenetic analysis based on chromosomal reversal, although most of the multi-*cagA H*. *pylori* strains clustered together, the NY40 and ELS37 hpEurope stains clustered close to hpAfrica1; this may suggest that the NY40 and ELS37 strains harbor genome structures similar to hpAfrica1 strains (Supplemental Fig. S1). Furthermore, clustering based on chromosomal arrangement of the 27 hpEurope strains revealed two distinct chromosomal structures in hpEurope: hpEurope/type-A and type-B. More importantly, the multi-*cagA* strains were only found in hpEurope/type-B (Fig. 2). Previous studies implied that hpEurope originated from two distinct ancestral populations: northeastern Africa and Central Asia^6,31^. However, efforts to define subpopulations of hpEurope were unsuccessful, suggesting a complex evolutionary history and extensive horizontal recombination events in hpEurope. Indeed, *H*. *pylori* is a highly recombinant bacterium and that signal of clonal inheritance was likely rapidly lost. In the current study, phylogeny inference based on chromosomal rearrangement was used to avoid this issue; this technique was powerful in that it was able to distinguish subsets of hpEurope. Additionally, completion and assembly of more complete genomes will help to confirm and extend our findings.

We previously identified strains carrying multiple copies of *cagA* using a PCR strategy that detects adjacent multiple *cagA* genes; our results were confirmed by Sanger sequencing and Southern blot analysis^16^. To investigate these strains in greater genomic detail, we chose PacBio technology for whole-genome sequencing of these multi-*cagA* genotype strains. We reasoned that because PacBio sequencing has a longer read length (~20 kb), it should be better for identification of the adjacent repeat sequences^32^. However, we were unable to assemble multiple *cagA* copies in the genomes of the strains known to carry multiple *cagA* copies (B125A, B128, B130A, B140, B147 and 7.13).

Since prior targeted Sanger sequencing showed that the *cagA* copy number in B125A, B128, B130A, B140, B147, 7.13 and J166 were at least two copies^16^, the difference between the genome assemblies and the targeted sequencing approaches may indicate limitations of the assembly programs or indicate that the dynamic expansion and contraction of *cagA* copy number occurs at a population level and consists of heterogenous population in terms of *cagA* gene number. Support of the later possibility can be found in our prior finding that *H*. *pylori* strain 7.13 is estimated to harbor an average *cagA* number of approximately 1.4^16^. Thus, genomic DNA for sequencing would have been isolated from heterogeneous populations of cells containing zero, one or two *cagA* copies; this issue could lead to issues with the downstream assembly process. Overall, these results suggest that many previous genome sequences may not reflect multiple copies of *cagA* that are actually present. Interestingly, among the analyzed sequences of 170 *cag*PAI positive strains available in the GenBank database, only the assemblies for strain PMSS1 and its derivative strain SS1 harbor more than one copy of the *cagA* gene. Further study will be required to examine the *cagA* gene numbers in other published type-B *cag*PAI strains since most of the next generation sequencing methods are not able to directly identify multiple *cagA* copies.

Previous analysis of heterogeneous population of strain PMSS1 strongly suggested that *H*. *pylori* can manipulate *cagA* number via homologous recombination within the CHA-ud repeat sequence^16^. In the current study, genome sequencing showed that the *cagA* genes in the multi-*cagA* strains were all flanked by the CHA-ud sequence. In addition, when either of the CHA-ud sequences that flank *cagA* were removed, PMSS1 lost the capability to generate the multi-*cagA* genotype. Previously expansion to multiple repeats was not detected in each single transformant of PMSS1/*cagA*-S^F^-1 and -S^L^-2 mutant strains^16^. In the current study, the prior observation of *cagA* duplication was confirmed by expanding the analysis to 200 individual colonies derived from each PMSS1 derivative (PMSS1/*cagA*-S^F^-1 and -S^L^-2). Furthermore, an additional PCR method was utilized to detect *cagA* deletion. Moreover, when G27 was engineered to contain CHA-ud or CHA-d sequences that flank *cagA* as direct repeats, G27 gained the ability to generate the multi-*cagA* genotype. This suggests that the CHA-ud genetic element flanking *cagA* is essential and sufficient for generation of the multi-*cagA* genotype; however, the CHA-ud sequence may not be unique for this capability; in theory any direct repeat that flanks *cagA* could result in a similar homologous recombination event. Indeed, introduction of flanking CHA-d sequences also resulted in the ability of G27 to duplicate *cagA* (Fig. 6). It is worth noting that G27/2CHA-ud and G27/2CHA-d strains carry approximately 1.1 and 1.3 copies of *cagA*, respectively (data not shown), while PMSS1 carries on average 3.7 copies of *cagA*^16^. The similar *cagA* copy number of G27/2CHA-ud and G27/2CHA-d strains to wild-type G27 might explain why there was no significant variation in the overall level of CagA expression in representative G27/revertant, G27/2CHA-ud and G27/2CHA-d strains as compared to that of wild-type G27 (Supplemental Fig. S5). Conversely, PMSS1 strains exhibited higher level of CagA expression than that of PMSS1/*cagA*-S^F^-1 and -S^L^-2 strains^16^.

The described genomic analyses lead us to propose a model whereby chromosomal rearrangements in the *cag*PAI region resulted in translocation and subsequently duplication of CHA-ud to flank *cagA*; this in turn leads to duplication of *cagA* (Fig. 7). In modern *H*. *pylori*, the *cag*PAI was thought to be derived from the most ancient *H*. *pylori*. However, our characterization of the *dg* region suggests that the *cag*PAI type-A and type-B strains evolved from two origins: pre-type-A and pre-type-B, respectively. The evolution of *cag*PAI type-A was similarly described by Fischer, but his model for *cag*PAI type-B evolution is different from our current model^33^. Data presented in Fig. 3 suggested that pre-type-B evolved separately from pre-type-A. Furthermore, our analysis (Fig. 5) suggests a role for the 3′ terminal region of a putative nonessential AAA family ATPase gene in pre-type-B strains in the origin of CHA-ud. Rearrangement within the *dg* region brought the putative ATPase gene to lie upstream of *cagA*, which successively became CHA-ud in all type-B strains. Next, CHA-ud duplicated to downstream of *cagA* probably through the activity of an IS element; IS elements were frequently present in this region^34^. It is worth noting that the phylogenetic analysis of chromosomal rearrangements using whole-genome sequences is based on the pattern of these rearrangements of co-linear blocks only for DNA regions that are shared by all of the strains. Thus, since some strains are *cag*PAI negative, the two types in hpEurope (Fig. 2) would not result from these two particular rearrangements in the *cag*PAI type-B strains. Together with the phylogenetic analyses, this model explains the distribution of multi-*cagA* strains only in Western hpEurope/type-B, but not in Western hpEurope/type-A and East Asian type *H*. *pylori*. From the observed data, it is likely that the evolution of the ATPase gene to become CHA-ud infrequently happened by a chromosomal reversal. However, since CagA plays an important role in bacterial pathogenesis, the multi-*cagA* genotype was retained. This strongly suggests that the multi-*cagA* genotype provided some evolutionary benefit to hpEurope/type-B and may do the same to other populations in the future.

## Materials and Methods

### Bacterial strains and culture

The *H*. *pylori* strains B125A, B128, B130A, B136A, B140, B147, J182, J166, 7.13, PMSS1, PMSS1/*cagA*-S^F^-1, PMSS1/*cagA*-S^L^-2, G27, and G27 isogenic mutant strains were cultured and stored as previously described^16,35^. Briefly, all *H*. *pylori* strains were grown on antibiotic supplemented Columbia blood agar plates under microaerobic conditions generated using an Anaeropack-Microaero gas-generating system (Mitsubishi Gas Chemical, Tokyo, Japan).

### Genome sequencing, assembly and annotation

Single colonies were isolated from B125A, B128, B130A, B136A, B140, B147, J182, 7.13. One single colony from each strain was further cultured for whole genome sequencing and preparation of a frozen stock. Bacterial genomic DNA was extracted using the Wizard® Genomic DNA Purification Kit (Promega, USA) and then Chunlab Inc. Korea processed the subsequent PacBio genome sequencing. Briefly, qualified genomic DNA was prepared as 20 kb SMRTbell templates using the SMRTbell Template Prep Kit (Pacific Biosciences, USA) according to the manufacturer’s protocol. The sequencing was performed using the PacBio RSII sequencing platform (Pacific Biosciences, USA) with the default condition for RSII P6C4 chemistry. The raw data were filtered and assembled with the SMRT Analysis Software v2.3.0 with the HGAP2 protocol and default parameters. The assembled genomes were processed for subsequent annotation by the NCBI Prokaryotic Genome Annotation Pipeline version 4.3^36^.

### Phylogenetic analysis

Phylogenetic analysis based on core genome pairwise distance was performed using Efficient Database framework for comparative Genome Analyses using BLAST score Ratios (EDGAR 2.0)^37^. Briefly, 927 orthologous genes that were shared among a total of 52 *H*. *pylori* genomes (Supplemental Table S1) were identified by bidirectional BLASTn^38^. Each of the orthologous genes was aligned with MUSCLE^39^. The alignments were then trimmed and concatenated to a single 855,682-bp alignment. The concatenated alignment was further used to compute a pairwise genome distance matrix; a phylogenetic tree was then constructed based on this distance matrix using a Neighbor-joining method as implemented in PHYLIP^40^. The Neighbor-joining tree was visualized using Mega 7.0^41^.

Phylogenetic analysis based on chromosomal inversion was computed using progressiveMauve and MGR^42,43^; all plasmids were first removed from the genome sequences. Briefly, the completed chromosomal sequences of the 46 general strains and 27 hpEurope strains were aligned with progressiveMauve using the default parameters; these resulted in 107 and 80 conserved local colinear blocks (LCBs), respectively. The relative position and orientation of the LCBs were output as signed permutations. Further, the permutations were used as input to MGR to compute phylogeny trees based on reversals with the unichromosomal circular mode. MGR generates a tree such that the sum of the rearrangements is minimized over all of the edges of the tree. Finally, the tree was midpoint-rooted and the tree topology was visualized using Mega 7.0.

### Generation of G27 isogenic mutant strains

Four constructs targeting the G27 region upstream of *cagA* were generated (Supplemental Fig. S4). Construct A was made as follows. Using the G27 wild type as a template, amplicons of front and rear wings with *Xho*I and *Sma*I sites located at the adjacent ends were generated using primer sets of T1cagAup1kbF and T1cagAup500RXS and G27ctrl5 and T1cagA0R, respectively. Both amplicons were fused by splicing-by-overlap-extension (SOE) PCR so that the fused amplicon contained *Xho*I and *Sma*I sites in the overlap-joining region^44,45^. This product was cloned into the pGEM®-T Easy vector system (Promega, Madison, WI, USA) by TA cloning. A *ksc*, liberated from plasmid pKSF-II by double-digestion with *Xho*I and *Sma*I, was subsequently ligated with the *Xho*I-*Sma*I double-digested plasmid that contained the fused amplicon^46,47^. Construct B was made using the primer set of T1cagAup1kbF and T1cagA0R, and the amplicon was directly cloned into the pGEM®-T Easy vector system. The three amplicons (starting from left to right) of construct C were made using the primer set of T1cagAup1kbF and CnDF2F1R, CnDF1F2F and T1cagAdown600R, and T1cagAup500F and T1cagA0R, respectively. The first two amplicons were fused together by SOE PCR, followed by the fusion of the third amplicon (rear wing) for making the complete insert; the resulting product was cloned into the pGEM®-T Easy vector system. Construct D was generated using the same process and primer sets of T1cagAup1kbF and CnEChaDF1R, CnEF1ChaDF and CnEF3ChaDR, and CnEChaDF3F and T1cagA0R. Three marker-less G27 isogenic mutant strains were generated in two sequential steps. At stage I, the construct A was introduced into G27 by natural transformation^48^. The successful transformants (G27/*ksc*) were isolated using kanamycin selection via growth on horse blood agar plates supplemented with 25 µg/mL kanamycin. The double homologous recombination of the construct, resulting in kanamycin-resistant *H*. *pylori* single colony isolates, were further verified by three PCRs (Aa, Ba, Ca, see Supplemental Fig. S4) and DNA sequencing. At stage II, constructs B, C and D were transformed into the previously selected G27*/ksc* strain for generation of the G27/revertant, G27/2CHA-ud and G27/2CHA-d respectively. The unmarked mutant strains derived from the intended double homologous recombination were isolated via growth on 5% sucrose supplemented horse blood agar; resulting colonies were verified by PCR screening (Ab, Bb, Cb; Ac, Bc, Cc; Ad, Bd, Cd) and DNA sequencing. The primers used for the construction and verification of the G27 mutant strains are listed in Supplemental Table S3.

### Genotyping of *cagA* copy number in *H*. *pylori* single colonies by colony PCR

Colony PCR was performed as previously described^16^. Briefly, a culture of PMSS1/*cagA*-S^F^-1, PMSS1/*cagA*-S^L^-2 or the G27 isogenic mutant strains was diluted in Brucella Broth and was plated onto horse blood agar plates. Plates were incubated for 4 days in microaerobic condition until single colonies appeared. Single colonies were picked and streaked onto a new blood agar plate for further culture. The cultures were used for isolation of genomic DNA and frozen stocks.

Colonies were transferred into 20 μL of distilled water and then were heated at 99 °C for 4 min. After centrifugation, the supernatant was used as the template for PCR to analyze *cagA* copy number in single colonies. A colony PCR was designed to identify none, single, or multiple *cagA* genes. The primers used for the *cagA* genotyping of the PMSS1 isogenic mutant strains and the G27 isogenic mutant strains are listed in Supplemental Table S3 and the alignment sites are illustrated in Fig. 6. The multiple *cagA* genotype was detected using the primer sets of dF2/dR2 for the PMSS1 isogenic mutants and G27dF2/dR2 for the G27 isogenic mutants. The *cagA* deletion was detected using the primer sets of cagAupF2/cagAdownR2 for the PMSS1 isogenic mutants and G27cagA1.1upF/cagAdownR2 for the G27 isogenic mutants. The presence of *cagA* was screened for using the F2 and G27R2 primer combination. Both *cagA* multiplication and deletion PCRs were performed as follows: 94 °C for 4 min; 35 cycles of 94 °C for 30 s, 54 °C for 15 s, and 72 °C for 90 s; a final step at 72 °C for 5 min. For the other PCR screening, the same PCR parameters, except a 30 s extension time, were used.

### Analysis of *H*. *pylori* chromosomal region between *dapB* and *glr* gene (*dg* region)

The annotated sequences were visualized using CLgenomics version 1.55 software with the format provided by Chunlab Inc., Korea. The strains used for the analysis of the *dg* region were downloaded from EzBioCloud (https://www.ezbiocloud.net/) with the accession numbers listed in Supplemental Table S2. Sequence homology was searched for using BLAST implemented in CLgenomics version 1.55. For strain B147, our prior Sanger dideoxy sequencing was unable to resolve the region upstream of *cagA*^16^; PacBio genomic sequencing revealed that a 39 kb insertion that was flanked by two integrases was responsible for the failed Sanger dideoxy sequencing (Fig. 4).

### DNA sequencing

Sanger dideoxy DNA sequencing was performed at Cosmo Genetech Co., Ltd (Seoul, Korea). The DNA sequencing primers are listed Supplemental Table S3.

### Accession numbers

All of the genome sequences were deposited to the NCBI GenBank Prokaryotic Genomes (https://www.ncbi.nlm.nih.gov/nucleotide/) under Bioproject: PRJNA419269. Sanger dideoxy sequencing results were deposited to the NCBI GenBank with accession numbers from MK089812 to MK089815.

### Immunoblot assay

To prepare lysates of infected cells, AGS cells were seeded onto 6-well cell culture plates at a density of 4 × 10^5^ cells per well and were then incubated for 2 days at 37 °C. At 2 h prior to infection, cells were washed with PBS and the medium was changed to 2 ml of RPMI 1640. Liquid cultures of *H*. *pylori* were resuspended in RPMI 1640, and AGS cells were infected at a MOI of 100 for 5 h. Subsequently, cells were washed with PBS and then lysed with 150 µl of cell lysis buffer supplemented with protease inhibitor cocktail. Protein concentrations of infected cell lysates were measured using Pierce bicinchoninic acid (BCA) protein assay reagent (Thermo Fisher Scientific, Waltham, MA, USA). 20 µg of each respective protein sample was separated by sodium dodecyl sulfate-polyacrylamide gel electrophoresis and then transferred to a polyvinylidene fluoride membrane (Merck Millipore, Darmstadt, Germany). To detect CagA, phosphorylated CagA, UreA, and GAPDH, membranes were probed by the use of 1:10,000 dilution of rabbit polyclonal anti-CagA antibody b-300 (Santa Cruz Biotechnology), 1:10,000 dilution of mouse monoclonal anti-phosphotyrosine antibody pY99 (Santa Cruz Biotechnology, Dallas, TX, USA), 1:3,000 rabbit polyclonal anti-Urease α antibody b-234 (Santa Cruz Biotechnology), and 1:2,500 rabbit polyclonal anti-GAPDH antibody (AbFrontier, Seoul, South Korea), respectively. These membranes were detected with 1:5,000 goat anti-mouse IgG-horseradish peroxidase (IgGHRP) (AbFrontier) or goat anti-rabbit IgG-HRP (AbFrontier). The pY99 treated membranes were stripped with Re-Blot Plus Strong Solution (Merck Millipore), prior to detection of total CagA. Probed membranes were then developed using Western Bright enhanced chemiluminescence-HRP (ECL-HRP) substrate (Advansta, Menlo Park, CA, USA) on X-ray film (Agfa, Mortsel, Belgium). Relative CagA expression levels were measured as previously described^16^. A ratio of CagA to UreA was calculated for each of five representative samples, and each value was normalized to the value of wild type G27. Next, the mean CagA expression levels from each group were divided with the G27/revertant value to determine relative CagA protein levels and those values were plotted on a graph. Error bars represent standard deviations from each group (n = 5).

## Supplementary information

Dataset 1

## Data Availability

All data generated or analyzed during this study are included in this published article (and its Supplementary Information files) or are available from the corresponding author on reasonable request.
