# Supplementary material for: Evolutionary mechanism leading to the multi-cagA genotype in Helicobacter pylori
Source: Sci Rep. 2019 Aug 1;9:11203. doi: 10.1038/s41598-019-47240-2 (PMC6672019; doi:10.1038/s41598-019-47240-2)
Supplement: Supplementary file 1 — Dataset 1 [file 41598_2019_47240_MOESM1_ESM.pdf]

Evolutionary mechanism leading to the multi-*cagA* genotype in *Helicobacter pylori*

Hanfu Su[a], Kavinda Tissera[b], Sungil Jang[b], Yun Hui Choi[b], Aeryun Kim[b], Yong-Joon Cho[c], Meiling Li[a], Niluka Gunawardhana[d], D. Scott Merrell[e], Linhu Ge[a]*, and Jeong-Heon Cha[a,b]*

[a] Key Laboratory of Oral Medicine, Guangzhou Institute of Oral Disease, Affiliated Stomatology Hospital of Guangzhou Medical University, Guangzhou, China

[b] Department of Oral Biology, Oral Science Research Center, Department of Applied Life Science, The Graduate School, BK21 Plus Project, Yonsei University College of Dentistry, Seoul, Republic of Korea

[c] Division of Polar Life Science, Korea Polar Research Institute, Incheon, Republic of Korea

[d] Department of Basic Sciences, Faculty of Dental Sciences, University of Peradeniya, Peradeniya, Sri Lanka

[e] Department of Microbiology and Immunology, Uniformed Services University of the Health Sciences, Bethesda, Maryland, USA

*Address correspondence to Linhu Ge, gelinhu@yeah.net or Jeong-Heon Cha, jcha@yuhs.ac.

H.S. and K.T. contributed equally to this article.

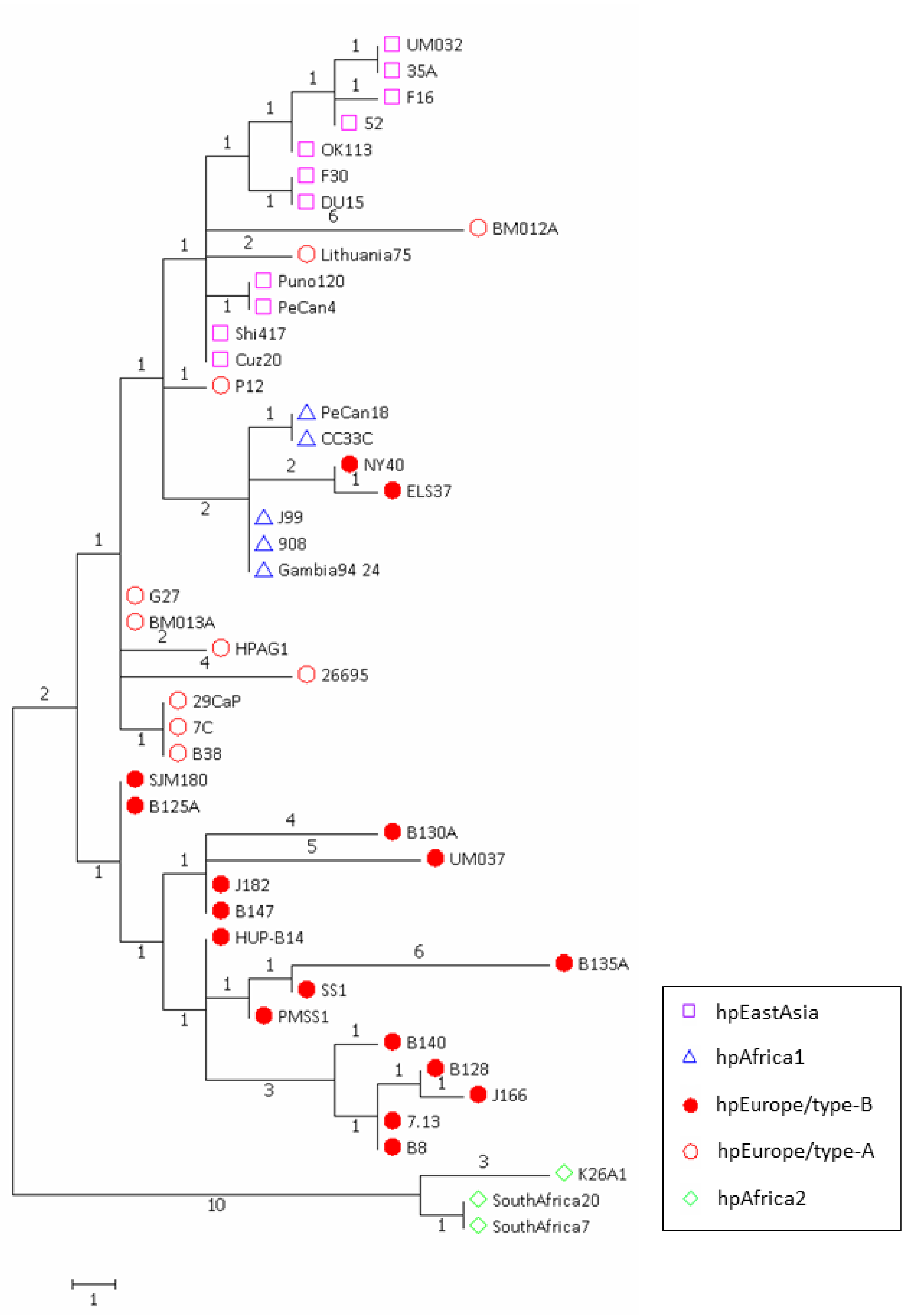

UM032
35A
F16
52
OK113
F30
DU15
BM012A
Lithuania75
Puno120
PeCan4
Shi417
Cuz20
P12
PeCan18
CC33C
NY40
ELS37
J99
908
Gambia94 24
G27
BM013A
HPAG1
26695
29CaP
7C
B38
SJM180
B125A
B130A
UM037
J182
B147
HUP-B14
B135A
SS1
PMSS1
B140
B128
J166
7.13
B8
K26A1
SouthAfrica20
SouthAfrica7
hpEastAsia
hpAfrica1
hpEurope/type-B
hpEurope/type-A
hpAfrica2

Supplemental Figure S1. Phylogenetic relationship of the 46 strains for which a complete genome was available. A total of 107 local colinear blocks from each of the 46 genomes were converted to a signed permutation. The signed permutation was further used to infer their phylogenetic relationship based on chromosomal reversals with a unichromosomal circular model. *H. pylori* populations are indicated. Scale bar indicates length of one rearrangement. The number of rearrangement is also indicated on each branch.

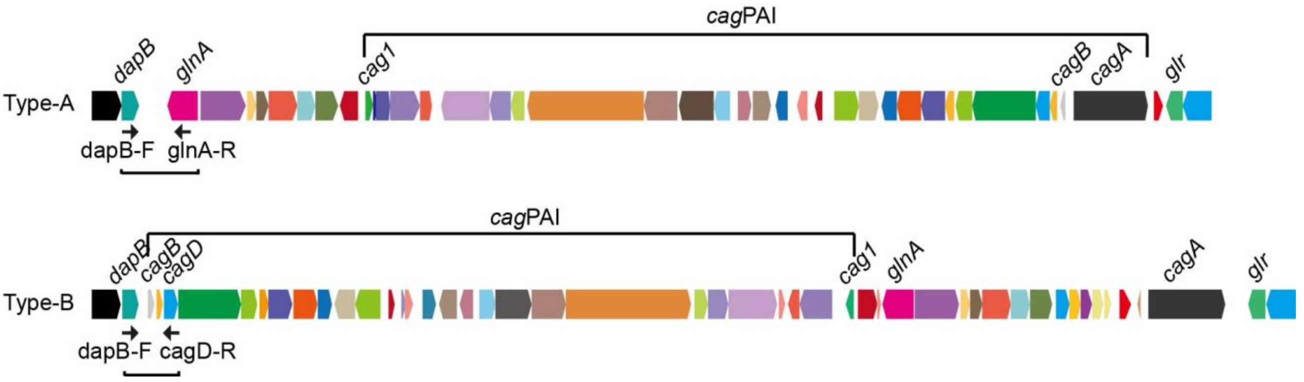

Supplemental Figure S2. *cag*PAI typing PCR. A typical gene arrangement of type-A and type-B is shown. PCR with primer sets of dapB-F / glnA-R and dapB-F / cagD-R were used to identify type-A and type-B, respectively. Primer annealing sites are indicated.

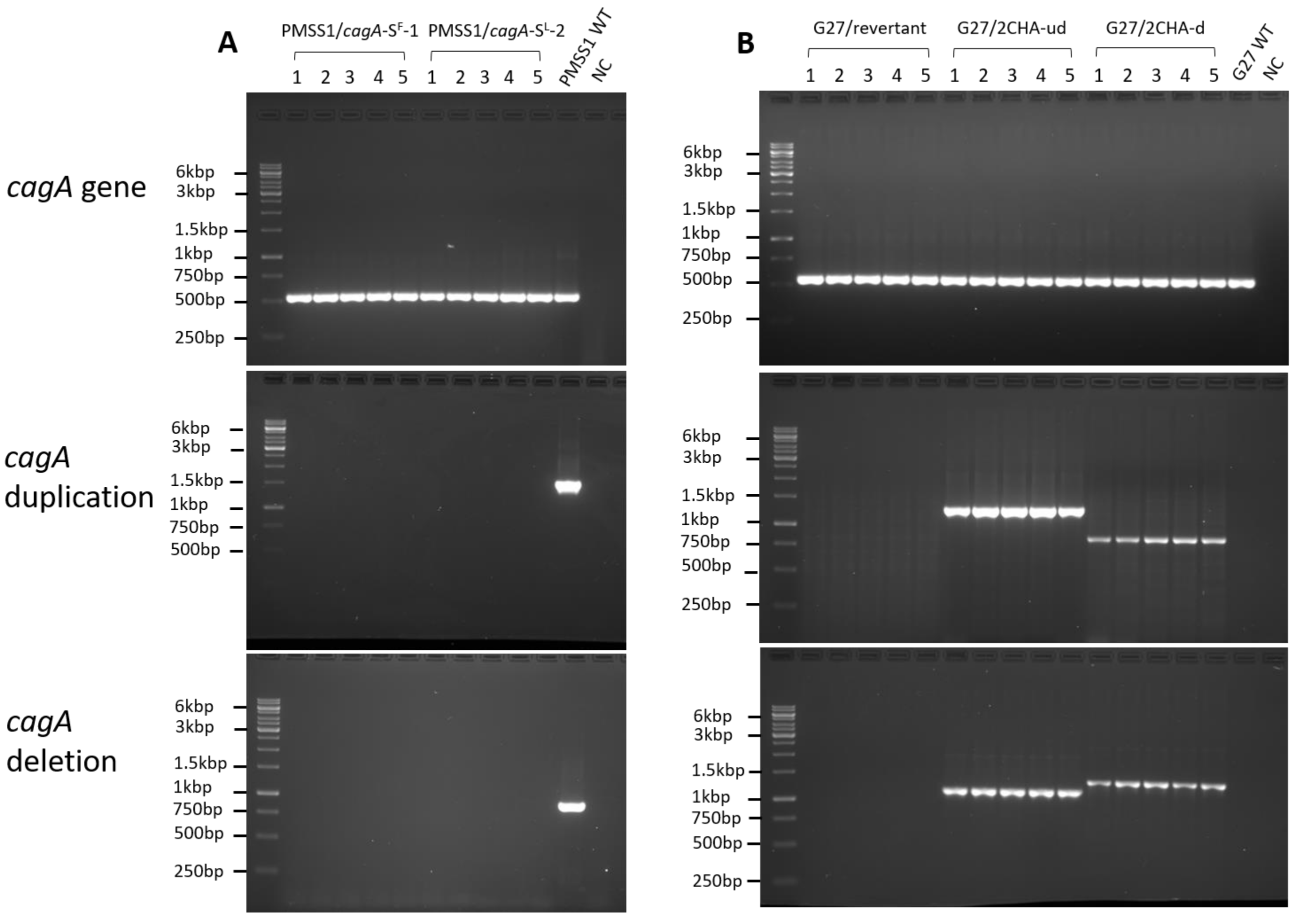

Supplemental Figure S3. PCR-based screening for multi-*cagA* genotypes of five representative single colony isolates from each PMSS1 and G27 isogenic mutant strains. A. Each of five representative single colony isolates derived from PMSS1/*cagA*-S^F-1 and PMSS1/*cagA*-S^L-2, containing only a single CHA-ud at either upstream or downstream of *cagA*, respectively, showed a PCR amplicon for *cagA* but no PCR amplicons for duplication and/or deletion of *cagA*. These data indicate a homogenous population carrying a single *cagA* gene. Conversely, wild-type PMSS1 showed PCR amplicons for *cagA*, *cagA* duplication and deletion, indicating a heterogenous population in terms of *cagA* copy number. Samples in each same group of *cagA* gene, *cagA* duplication, and *cagA* deletion were loaded onto same gel and shown, respectively. B. Each of five representative single colony isolates of G27/2CHA-ud and G27/2CHA-d showed PCR amplicons for *cagA*, duplication and deletion, indicating a heterogenous population in terms of *cagA* copy number. Conversely, the G27/revertant and wild-type G27 showed PCR amplicon for *cagA* but did not show any PCR amplicons for duplication and/or deletion of *cagA*. These data indicate a homogenous population of single copy of

cagA. Primers were indicated in Figure 6. Samples in each same group of *cagA* gene, *cagA* duplication, and *cagA* deletion were loaded onto same gel and shown, respectively.

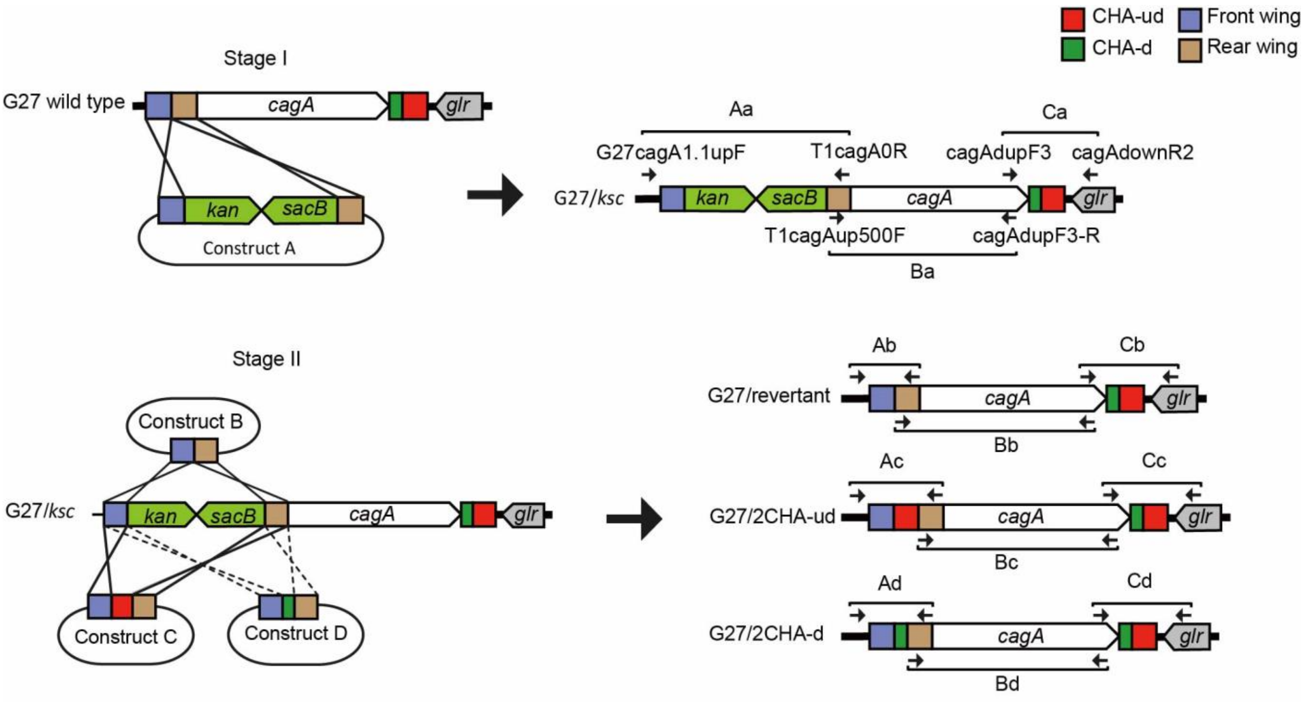

Supplemental Figure S4. Illustration of the generation of G27 isogenic mutant strains. Construct A, B, C, and D were used to generate targeted isogenic mutant strains. Double crossover regions are shown. PCR primers that were used for confirmation are also indicated.

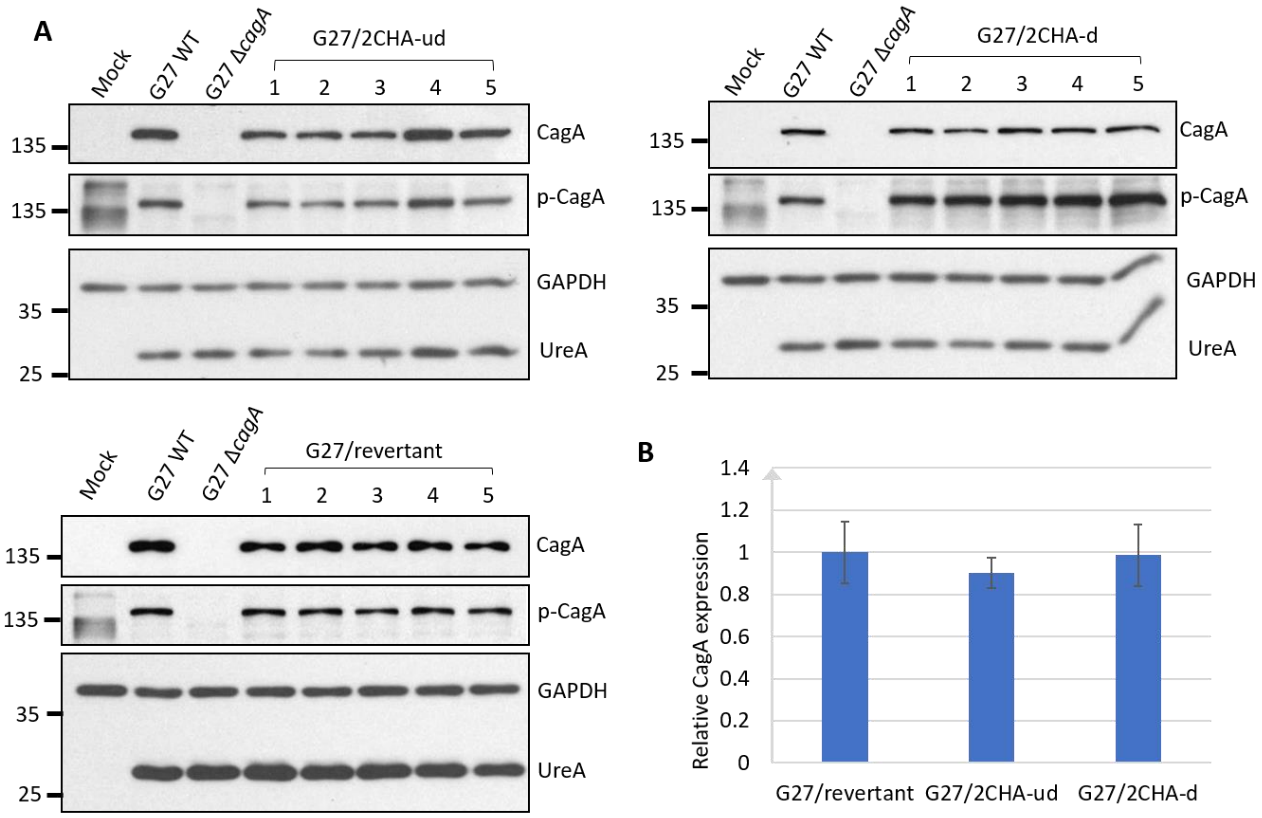

Supplemental Figure S5. Relative CagA expression in G27 mutant strains. A. Lysates of AGS cells that were infected with each of five representative *H. pylori* single colony isolates of G27/2CHA-ud, G27/2CHA-d and G27/revertant were immunoblotted for CagA and phosphorylated CagA (p-CagA) as well as glyceraldehyde-3-phosphate dehydrogenase (GAPDH) and UreA as controls. Samples in each same group of G27 mutants as well as controls were loaded onto same gel and transferred to same membrane; membranes were cut into upper and lower sides for blotting of CagA/p-CagA and GAPDH/UreA, respectively; CagA bands were generated from the same blot after stripping p-CagA antibody and re-probing. Molecular weight markers (kD) were indicated on the left. The bands were acquired from film scans shown in Supplemental Figure S6. B. Relative CagA protein levels of G27/revertant, G27/2CHA-ud and G27/2CHA-d were determined. There was no significant difference for CagA expression. Error bars represent standard deviations.

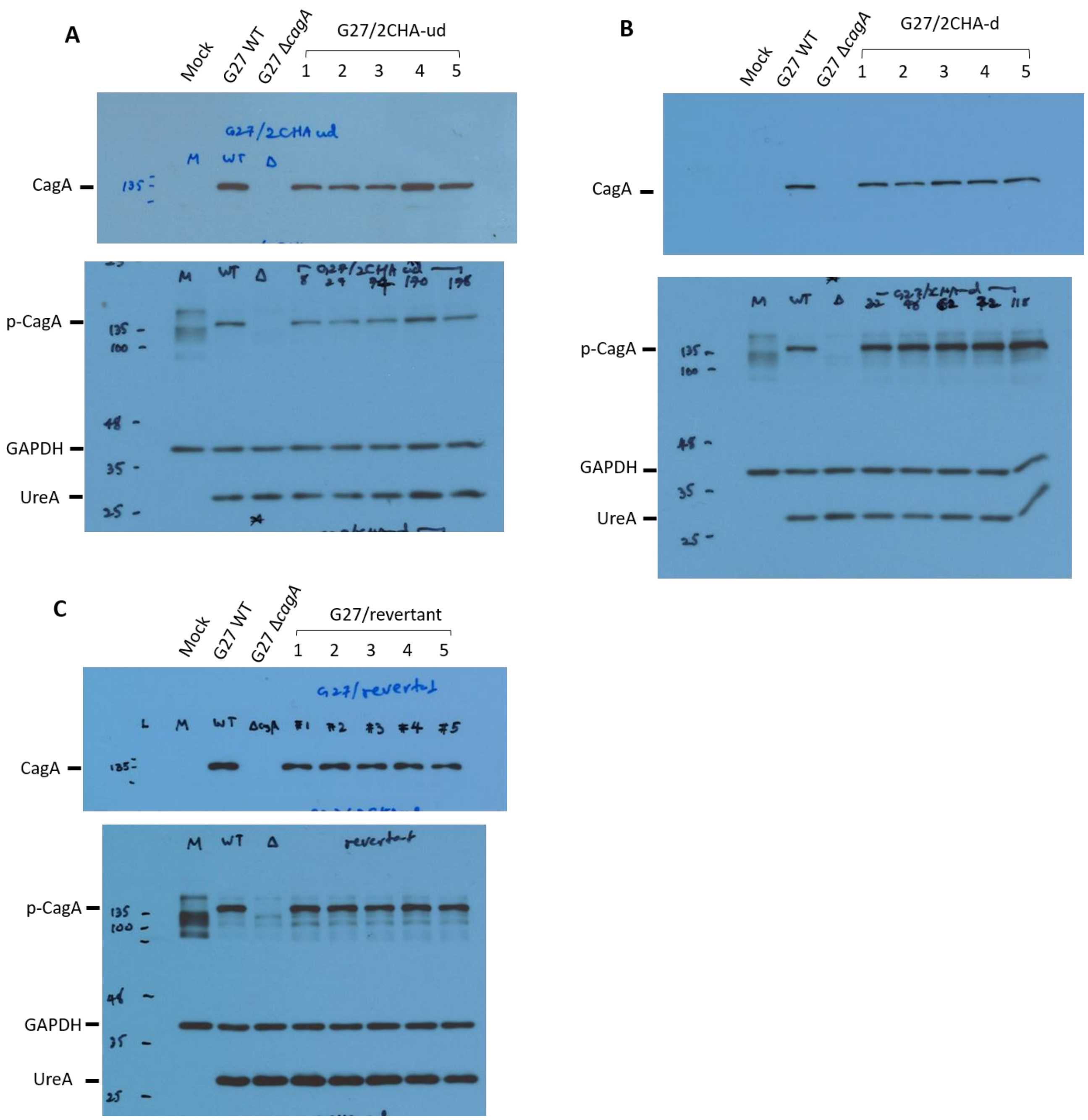

Supplemental Figure S6. Full length Western blot scans utilized in Supplemental Figure S5.

A. G27/2CHA-ud. B. G27/2CHA-d. C. G27/revertant.

Supplemental Table S1. List of *Helicobacter pylori* strains and their accession numbers used for phylogenetic analysis in Fig. 1.

Helicobacter pylori strains	accession number
G27	GCA_000021165.1
P12	GCA_000021465.1
Lithuania75	GCA_000185225.1
HPAG1	GCA_000013245.1
26695	GCA_000008525.1
B38	GCA_000091345.1
BM012A	GCA_000498315.1
BM013A	GCA_000685665.1
7C	GCA_001433515.1
29CaP	GCA_001433495.1
NY40	GCA_000828955.1
SJM180	GCA_000148855.1
HUP-B14	GCA_000259235.1
ELS37	GCA_000255955.1
UM037	GCA_000392515.3
J166	GCA_000685625.1
PMSS1	GCA_001991095.1
SS1	GCA_002005525.1
B8	GCA_000196755.1
H-11	GCA_000274485.2
UM045	GCA_000401395.1
NAD1	GCA_000256035.2
N6	GCA_000285895.1
59	GCA_001278525.1
NQ4216	GCA_000274945.2
SouthAfrica7	GCA_000185245.1
SouthAfrica20	GCA_000590775.1
K26A1	GCA_001653455.1
PeCan18	GCA_000277425.1
908	GCA_000148665.1
Gambia94-24	GCA_000185205.1
J99	GCA_000008785.1
CC33C	GCA_001653415.1
PeCan4	GCA_000148875.1
Shi417	GCA_000277365.1
Cuz20	GCA_000148895.1

Puno120	GCA_000224535.1
F16	GCA_000270005.1
F30	GCA_000270025.1
DU15	GCA_001653395.1
52	GCA_000023805.1
UM032	GCA_000392455.3
OK113	GCA_000348865.1
35A	GCA_000178935.2
B125A	this study
B130A	this study
B136A	this study
B140	this study
B128	this study
7.13	this study
J182	this study
B147	this study

Supplemental Table S2. List of *Helicobacter pylori* strains and their accession numbers used for *cag*PAI analysis.

Organism/Name	Strain	Assembly
"Helicobacter pylori 26695"	"26695"	GCA_000008525.1
"Helicobacter pylori 51"	"51"	GCA_000011725.1
"Helicobacter pylori HPAG1"	"HPAG1"	GCA_000013245.1
"Helicobacter pylori Shi470"	"Shi470"	GCA_000020245.1
"Helicobacter pylori G27"	"G27"	GCA_000021165.1
"Helicobacter pylori P12"	"P12"	GCA_000021465.1
"Helicobacter pylori 52"	"52"	GCA_000023805.1
"Helicobacter pylori B38"	"B38"	GCA_000091345.1
"Helicobacter pylori v225d"	"v225d"	GCA_000093185.1
"Helicobacter pylori 908"	"908"	GCA_000148665.1
"Helicobacter pylori SJM180"	"SJM180"	GCA_000148855.1
"Helicobacter pylori PeCan4"	"PeCan4"	GCA_000148875.1
"Helicobacter pylori Cuz20"	"Cuz20"	GCA_000148895.1
"Helicobacter pylori Sat464"	"Sat464"	GCA_000148915.1
"Helicobacter pylori 35A"	"35A"	GCA_000178935.2
"Helicobacter pylori India7"	"India7"	GCA_000185185.1
"Helicobacter pylori Gambia94/24"	"Gambia94/24"	GCA_000185205.1
"Helicobacter pylori Lithuania75"	"Lithuania75"	GCA_000185225.1
"Helicobacter pylori SouthAfrica7"	"SouthAfrica7"	GCA_000185245.1

"Helicobacter pylori B8"	"B8"	GCA_000196755.1
"Helicobacter pylori 83"	"83"	GCA_000213135.1
"Helicobacter pylori Puno120"	"Puno120"	GCA_000224535.1
"Helicobacter pylori Puno135"	"Puno135"	GCA_000224555.1
"Helicobacter pylori SNT49"	"Santal49"	GCA_000224575.1
"Helicobacter pylori ELS37"	"ELS37"	GCA_000255955.1
"Helicobacter pylori HUP-B14"	"HUP-B14"	GCA_000259235.1
"Helicobacter pylori XZ274"	"XZ274"	GCA_000262655.1
"Helicobacter pylori F16"	"F16"	GCA_000270005.1
"Helicobacter pylori F30"	"F30"	GCA_000270025.1
"Helicobacter pylori F32"	"F32"	GCA_000270045.1
"Helicobacter pylori F57"	"F57"	GCA_000270065.1
"Helicobacter pylori Shi417"	"Shi417"	GCA_000277365.1
"Helicobacter pylori Shi169"	"Shi169"	GCA_000277385.1
"Helicobacter pylori Shi112"	"Shi112"	GCA_000277405.1
"Helicobacter pylori PeCan18"	"PeCan18"	GCA_000277425.1
"Helicobacter pylori Aklavik117"	"Aklavik117"	GCA_000315955.1
"Helicobacter pylori Aklavik86"	"Aklavik86"	GCA_000317875.1
"Helicobacter pylori OK113"	"OK113"	GCA_000348865.1
"Helicobacter pylori OK310"	"OK310"	GCA_000348885.1
"Helicobacter pylori UM032"	"UM032"	GCA_000392455.3
"Helicobacter pylori UM299"	"UM299"	GCA_000392475.3
"Helicobacter pylori UM037"	"UM037"	GCA_000392515.3
"Helicobacter pylori UM066"	"UM066"	GCA_000392535.3
"Helicobacter pylori UM298"	"UM298"	GCA_000439295.2
"Helicobacter pylori BM012A"	"BM012A"	GCA_000498315.1
"Helicobacter pylori BM012S"	"BM012S"	GCA_000498335.1
"Helicobacter pylori SouthAfrica20"	"SouthAfrica20"	GCA_000590775.1
"Helicobacter pylori oki102"	"oki102"	GCA_000600045.1
"Helicobacter pylori oki112"	"oki112"	GCA_000600085.1
"Helicobacter pylori oki128"	"oki128"	GCA_000600125.1
"Helicobacter pylori oki154"	"oki154"	GCA_000600145.1
"Helicobacter pylori oki422"	"oki422"	GCA_000600165.1
"Helicobacter pylori oki673"	"oki673"	GCA_000600185.1
"Helicobacter pylori oki828"	"oki828"	GCA_000600205.1
"Helicobacter pylori oki898"	"oki898"	GCA_000600225.1
"Helicobacter pylori J166"	"J166"	GCA_000685625.1
"Helicobacter pylori BM013A"	"BM013A"	GCA_000685665.1
"Helicobacter pylori BM012B"	"BM012B"	GCA_000685705.1
"Helicobacter pylori BM013B"	"BM013B"	GCA_000685745.1
"Helicobacter pylori Hp238"	"Hp238"	GCA_000817025.1

"Helicobacter pylori NY40"	"NY40"	GCA_000828955.1
"Helicobacter pylori J99"	"J99"	GCA_000982695.1
"Helicobacter pylori 29CaP"	"29CaP"	GCA_001433495.1
"Helicobacter pylori 7C"	"7C"	GCA_001433515.1
"Helicobacter pylori ML1"	"ML1"	GCA_001549715.1
"Helicobacter pylori ML3"	"ML3"	GCA_001549875.1
"Helicobacter pylori GAM100Ai"	"GAM100Ai"	GCA_000310005.2
"Helicobacter pylori GAM101Biv"	"GAM101Biv"	GCA_000344945.2
"Helicobacter pylori GAM103Bi"	"GAM103Bi"	GCA_000344965.2
"Helicobacter pylori GAM105Ai"	"GAM105Ai"	GCA_000344985.2
"Helicobacter pylori GAM112Ai"	"GAM112Ai"	GCA_000345005.2
"Helicobacter pylori GAM114Ai"	"GAM114Ai"	GCA_000345025.2
"Helicobacter pylori GAM115Ai"	"GAM115Ai"	GCA_000345045.2
"Helicobacter pylori GAM201Ai"	"GAM201Ai"	GCA_000345065.2
"Helicobacter pylori GAM118Bi"	"GAM118Bi"	GCA_000345085.2
"Helicobacter pylori GAM119Bi"	"GAM119Bi"	GCA_000345105.2
"Helicobacter pylori GAM120Ai"	"GAM120Ai"	GCA_000345125.2
"Helicobacter pylori GAM121Aii"	"GAM121Aii"	GCA_000345145.2
"Helicobacter pylori GAM210Bi"	"GAM210Bi"	GCA_000345165.2
"Helicobacter pylori GAM231Ai"	"GAM231Ai"	GCA_000345185.2
"Helicobacter pylori GAM239Bi"	"GAM239Bi"	GCA_000345205.2
"Helicobacter pylori GAM244Ai"	"GAM244Ai"	GCA_000345225.2
"Helicobacter pylori GAM245Ai"	"GAM245Ai"	GCA_000345245.2
"Helicobacter pylori GAM246Ai"	"GAM246Ai"	GCA_000345265.2
"Helicobacter pylori GAM249T"	"GAM249T"	GCA_000345285.2
"Helicobacter pylori GAM250AFi"	"GAM250AFi"	GCA_000345305.2
"Helicobacter pylori GAM250T"	"GAM250T"	GCA_000345325.2
"Helicobacter pylori GAM252Bi"	"GAM252Bi"	GCA_000345345.2
"Helicobacter pylori GAM252T"	"GAM252T"	GCA_000345365.2
"Helicobacter pylori GAM254Ai"	"GAM254Ai"	GCA_000345385.2
"Helicobacter pylori GAM260ASi"	"GAM260ASi"	GCA_000345405.2
"Helicobacter pylori GAM260Bi"	"GAM260Bi"	GCA_000345425.2
"Helicobacter pylori GAM260BSi"	"GAM260BSi"	GCA_000345445.2
"Helicobacter pylori GAM263BFi"	"GAM263BFi"	GCA_000345465.2
"Helicobacter pylori GAM264Ai"	"GAM264Ai"	GCA_000345485.2
"Helicobacter pylori GAM265BSii"	"GAM265BSii"	GCA_000345505.2
"Helicobacter pylori GAM268Bii"	"GAM268Bii"	GCA_000345525.2
"Helicobacter pylori GAM270ASi"	"GAM270ASi"	GCA_000345545.2
"Helicobacter pylori GAM42Ai"	"GAM42Ai"	GCA_000345565.2
"Helicobacter pylori GAM71Ai"	"GAM71Ai"	GCA_000345585.2
"Helicobacter pylori GAM80Ai"	"GAM80Ai"	GCA_000345605.2

"Helicobacter pylori GAM83Bi"	"GAM83Bi"	GCA_000345625.2
"Helicobacter pylori GAM83T"	"GAM83T"	GCA_000345645.2
"Helicobacter pylori GAM93Bi"	"GAM93Bi"	GCA_000345665.2
"Helicobacter pylori GAM96Ai"	"GAM96Ai"	GCA_000345685.2
"Helicobacter pylori GAMchJs106B"	"GAMchJs106B"	GCA_000345705.2
"Helicobacter pylori HP116Bi"	"HP116Bi"	GCA_000345725.2
"Helicobacter pylori HP250AFii"	"HP250AFii"	GCA_000345745.2
"Helicobacter pylori HP250AFiii"	"HP250AFiii"	GCA_000345765.2
"Helicobacter pylori HP250AFiV"	"HP250AFiV"	GCA_000345785.2
"Helicobacter pylori HP250ASi"	"HP250ASi"	GCA_000345805.2
"Helicobacter pylori HP250ASii"	"HP250ASii"	GCA_000345825.2
"Helicobacter pylori HP250BFi"	"HP250BFi"	GCA_000345845.2
"Helicobacter pylori HP250BFii"	"HP250BFii"	GCA_000345865.2
"Helicobacter pylori HP250BFiii"	"HP250BFiii"	GCA_000345885.2
"Helicobacter pylori HP250BFiV"	"HP250BFiV"	GCA_000345905.2
"Helicobacter pylori HP250BSi"	"HP250BSi"	GCA_000345925.2
"Helicobacter pylori HP260AFi"	"HP260AFi"	GCA_000345945.2
"Helicobacter pylori HP260AFii"	"HP260AFii"	GCA_000345965.2
"Helicobacter pylori HP260ASii"	"HP260ASii"	GCA_000345985.2
"Helicobacter pylori HP260BFii"	"HP260BFii"	GCA_000346005.2
"Helicobacter pylori HP260Bi"	"HP260Bi"	GCA_000346025.2
"Helicobacter pylori GAMchJs114i"	"GAMchJs114i"	GCA_000346815.2
"Helicobacter pylori GAMchJs117Ai"	"GAMchJs117Ai"	GCA_000346835.2
"Helicobacter pylori GAMchJs124i"	"GAMchJs124i"	GCA_000346855.2
"Helicobacter pylori GAMchJs136i"	"GAMchJs136i"	GCA_000346875.2
"Helicobacter pylori B45"	"B45"	GCA_000234645.3
"Helicobacter pylori HP2RS"	"HP2RS"	GCA_000258825.1
"Helicobacter pylori CCUG 17874"	"CCUG 17874"	GCA_000258845.1
"Helicobacter pylori Hp H-30"	"Hp H-30"	GCA_000273865.2
"Helicobacter pylori Hp H-45"	"Hp H-45"	GCA_000273945.1
"Helicobacter pylori Hp H-43"	"Hp H-43"	GCA_000273965.1
"Helicobacter pylori Hp A-14"	"Hp A-14"	GCA_000274005.2
"Helicobacter pylori Hp A-26"	"Hp A-26"	GCA_000274025.1
"Helicobacter pylori Hp A-27"	"Hp A-27"	GCA_000274045.2
"Helicobacter pylori Hp H-9"	"Hp H-9"	GCA_000274085.2
"Helicobacter pylori Hp P-11"	"Hp P-11"	GCA_000274205.2
"Helicobacter pylori Hp P-16"	"Hp P-16"	GCA_000274305.1
"Helicobacter pylori Hp P-23"	"Hp P-23"	GCA_000274325.1

"Helicobacter pylori Hp P-74"	"Hp P-74"	GCA_000274345.1
"Helicobacter pylori Hp P-11b"	"Hp P-11b"	GCA_000274385.2
"Helicobacter pylori Hp P-15b"	"Hp P-15b"	GCA_000274425.2
"Helicobacter pylori Hp P-1b"	"Hp P-1b"	GCA_000274445.2
"Helicobacter pylori Hp H-11"	"Hp H-11"	GCA_000274485.2
"Helicobacter pylori NQ4161"	"NQ4161"	GCA_000274525.2
"Helicobacter pylori NQ4110"	"NQ4110"	GCA_000274545.2
"Helicobacter pylori NQ4099"	"NQ4099"	GCA_000274565.1
"Helicobacter pylori NQ4053"	"NQ4053"	GCA_000274605.1
"Helicobacter pylori CPY6311"	"CPY6311"	GCA_000274645.2
"Helicobacter pylori Hp P-8b"	"Hp P-8b"	GCA_000274765.2
"Helicobacter pylori Hp P-3"	"Hp P-3"	GCA_000274825.2
"Helicobacter pylori Hp P-4"	"Hp P-4"	GCA_000274845.2
"Helicobacter pylori Hp M3"	"Hp M3"	GCA_000274885.2
"Helicobacter pylori NQ4216"	"NQ4216"	GCA_000274945.2
"Helicobacter pylori Hp M1"	"Hp M1"	GCA_000274965.2
"Helicobacter pylori Hp M2"	"Hp M2"	GCA_000274985.2
"Helicobacter pylori Hp M6"	"Hp M6"	GCA_000275005.2
"Helicobacter pylori Hp M5"	"Hp M5"	GCA_000275025.2
"Helicobacter pylori Hp H-27"	"Hp H-27"	GCA_000275105.1
"Helicobacter pylori Hp H-28"	"Hp H-28"	GCA_000275125.1
"Helicobacter pylori Hp P-25d"	"Hp P-25d"	GCA_000275145.2
"Helicobacter pylori CPY1124"	"CPY1124"	GCA_000275225.2
"Helicobacter pylori Hp H-16"	"Hp H-16"	GCA_000275245.2
"Helicobacter pylori Hp A-17"	"Hp A-17"	GCA_000275265.2
"Helicobacter pylori Hp A-4"	"Hp A-4"	GCA_000275305.2
"Helicobacter pylori Hp P-28b"	"Hp P-28b"	GCA_000275345.2
"Helicobacter pylori Hp P-4c"	"Hp P-4c"	GCA_000275365.2
"Helicobacter pylori Hp P-30"	"Hp P-30"	GCA_000275405.2
"Helicobacter pylori Hp P-62"	"Hp P-62"	GCA_000275445.2
"Helicobacter pylori Hp M9"	"Hp M9"	GCA_000275485.2
"Helicobacter pylori Hp P-2b"	"Hp P-2b"	GCA_000275505.2
"Helicobacter pylori Hp P-3b"	"Hp P-3b"	GCA_000275525.2
"Helicobacter pylori Hp M4"	"Hp M4"	GCA_000275545.2
"Helicobacter pylori Hp H-24c"	"Hp H-24c"	GCA_000275565.2
"Helicobacter pylori Hp H-24b"	"Hp H-24b"	GCA_000275585.2
"Helicobacter pylori N6"	"N6"	GCA_000285895.1
"Helicobacter pylori R036d"	"R036d"	GCA_000299715.1
"Helicobacter pylori R038b"	"R038b"	GCA_000299755.1
"Helicobacter pylori R055a"	"R055a"	GCA_000299795.1
"Helicobacter pylori R056a"	"R056a"	GCA_000299815.1

"Helicobacter pylori R32b"	"R32b"	GCA_000299835.1
"Helicobacter pylori R018c"	"R018c"	GCA_000299855.1
"Helicobacter pylori A45"	"A45"	GCA_000333835.1
"Helicobacter pylori UMB_G1"	"UMB_G1"	GCA_000349465.1
"Helicobacter pylori UM045"	"UM045"	GCA_000401395.1
"Helicobacter pylori wls-5-12"	"wls-5-12"	GCA_000444195.2
"Helicobacter pylori HP87tlpD"	"HP87tlpD"	GCA_000469985.1
"Helicobacter pylori HP87P7tlpDRI"	"HP87tlpDRI"	GCA_000470075.1
"Helicobacter pylori HP87hu"	"HP87hu"	GCA_000470135.1
"Helicobacter pylori HP87P7"	"HP87P7"	GCA_000470355.1
"Helicobacter pylori SA302C"	"SA302C"	GCA_000475515.1
"Helicobacter pylori SA171A"	"SA171A"	GCA_000476055.1
"Helicobacter pylori SA302A"	"SA302A"	GCA_000476175.1
"Helicobacter pylori SA173A"	"SA173A"	GCA_000476575.1
"Helicobacter pylori SA172A"	"SA172A"	GCA_000476595.1
"Helicobacter pylori SA171C"	"SA171C"	GCA_000476735.1
"Helicobacter pylori SA221C"	"SA221C"	GCA_000476855.1
"Helicobacter pylori SA165A"	"SA165A"	GCA_000476915.1
"Helicobacter pylori SA165C"	"SA165C"	GCA_000477075.1
"Helicobacter pylori SA164A"	"SA164A"	GCA_000477115.1
"Helicobacter pylori SA164C"	"SA164C"	GCA_000477215.1
"Helicobacter pylori X47-2AL"	"X47-2AL"	GCA_000497465.2
"Helicobacter pylori Sahul64"	"Sahul64"	GCA_000513515.1
"Helicobacter pylori H3014"	"H3014"	GCA_000824865.1
"Helicobacter pylori H3016"	"H3016"	GCA_000824885.1
"Helicobacter pylori H3018"	"H3018"	GCA_000824925.1
"Helicobacter pylori UM139"	"UM139"	GCA_001185015.1
"Helicobacter pylori UM029"	"UM209"	GCA_001189655.1
"Helicobacter pylori UM211"	"UM211"	GCA_001189735.1
"Helicobacter pylori UM147"	"UM147"	GCA_001189835.1
"Helicobacter pylori 22"	"22"	GCA_001278515.1
"Helicobacter pylori 59"	"59"	GCA_001278525.1
"Helicobacter pylori TC-2_10"	"TC-2_10"	GCA_900087515.1
"Helicobacter pylori 1152/04"	"1152/04"	GCA_000802445.1
"Helicobacter pylori 1089/03"	"1089/03"	GCA_000802455.1
"Helicobacter pylori 1198/04"	"1198/04"	GCA_000802465.1
"Helicobacter pylori 207/99"	"207/99"	GCA_000802505.1
"Helicobacter pylori 499/02"	"499/02"	GCA_000802525.1
"Helicobacter pylori 228/99"	"228/99"	GCA_000802545.1
"Helicobacter pylori 655/99"	"655/99"	GCA_000802575.1

"Helicobacter pylori 1786/05"	"1786/05"	GCA_000802605.1
"Helicobacter pylori 173/00"	"173/00"	GCA_000802625.1
"Helicobacter pylori 1846/05"	"1846/05"	GCA_000802645.1
"Helicobacter pylori UM276S"	"UM276S"	GCA_001446295.2
"Helicobacter pylori H3017"	"H3017"	GCA_000824905.1
"Helicobacter pylori JCM 12093"	"JCM 12093"	GCA_001312765.1
"Helicobacter pylori L7"	"L7"	GCA_001653375.1
"Helicobacter pylori DU15"	"DU15"	GCA_001653395.1
"Helicobacter pylori CC33C"	"CC33C"	GCA_001653415.1
"Helicobacter pylori ausabrJ05"	"ausabrJ05"	GCA_001653435.1
"Helicobacter pylori K26A1"	"K26A1"	GCA_001653455.1
"Helicobacter pylori NAD1"	"NAD1"	GCA_000256035.2
"Helicobacter pylori PNG84A"	"PNG84A"	GCA_001653475.1
"Helicobacter pylori PMSS1"	"PMSS1"	GCA_001991095.1
"Helicobacter pylori SS1"	"SS1"	GCA_002005525.1
"Helicobacter pylori B147"	B147	this study
"Helicobacter pylori B125A"	B125A	this study
"Helicobacter pylori B128"	B128	this study
"Helicobacter pylori B130A"	B130A	this study
"Helicobacter pylori B136A"	B136A	this study
"Helicobacter pylori B140"	B140	this study
"Helicobacter pylori J182"	J182	this study
"Helicobacter pylori 7.13"	7.13	this study

Supplemental Table S3. Primers used in this study.

Primer name	Sequence (5′→3′)	Description
T1cagAup1kbF[p]	CGC TAG AAC AGA AAT GAT CTG	Forward primer binding at approximately 1kbp (954-974) upstream of G27 *cagA* gene.
T1cagAup500RXS[p]	CCC GGG AGG CTC GAG GAG TTG CTT AAA ATG GAG C	Fusion reverse primer with XhoI SmaI site at the 5' end, which binds at arround 500bp upstream of G27 *cagA* gene.
G27ctrl5[p]	CTC GAG CCT CCC GGG CA TAG ACC ACT AAA GAA AC	Fusion forward primer with XhoI SmaI site at the 5' end, which binds at roughly 500bp upstream of G27 *cagA* gene.
T1cagA0R[p,q]	TTC GTT AGT CAT TGT TTC TC	Reverse primer annealing at G27 cagA including the upstream region (orf -8 to 12bp)
CnDF2F1R[p]	GTT TTT TCT AGT TGA TGC TCG AGT TGC TTA AAA TGG AGC T	Fusion reverse primer annealing at arround 500bp upstream of *cagA*. 20bp present at the 5' region belongs to the CHA-ud.
CnDF1F2F[p,r]	AGC TCC ATT TTA AGC AAC TCG AGC ATC AAC TAG AAA AAA CAA G	Fusion forward primer which is complementary to CnDF2F1R
T1cagAdown600R[p]	GTT TCT TTA GTG GTC TAT GAA GGG TAT TGT GTT GAT TAT AA	Fusion reverse primer which binds at arround 600bp downstream of G27 *cagA* gene
T1cagAup500F[p,q]	TTA TAA TCA ACA CAA TAC CCT TCA TAG ACC ACT AAA GAA AC	Fusion forward primer which is complementary to T1cagAdown600R
CnEChaDF1R[p]	GTT AAA AAA CCC CTT GTT TTT AGA GTT GCT TAA AAT GGA GCT	Fusion reverse primer annealing at roughly 500bp upstream of *cagA*. 21bp present at the 5' region belongs to the CHA-d.
CnEF1ChaDF[p,r]	AGC TCC ATT TTA AGC AAC TCT AAA AAC AAG GGG TTT TTT AAC	Fusion forward primer which is complementary to CnEChaDF1R
CnEF3ChaDR[p]	GTT TCT TTA GTG GTC TAT GTC TTT ATA ACC CTT AAT TAA TTC C	Fusion reverse primer binds at CHA-d 500bp upstream of *cagA*. 21bp present at the 5' region belongs to the CHA-d.
CnEChaDF3F[p]	TTA ATT AAG GGT TAT AAA GAC ATA GAC CAC TAA AGA AAC	Fusion forward primer which is complementary to CnEF3ChaDR
G27cagA1.1upF[q,r,s,t]	GCG CGT AAG CAA AAA CAG TC	Forward primer binds to the 1.1kbp upstream of the G27 *cagA*
cagAdupF3-R[q]	GAA TCA GAA TAA TCT TTC ATA	Reverse primer annealing in G27 cagA orf 3465-3485bp
cagAdupF3[q]	TAT GAA AGA TTA TTC TGA TTC	Forward primer annealing in G27 *cagA* orf 3465-3485bp
cagAdownR2[q,s,t,u]	GCA CAC GCA TTC CCT AAA GT	Reverse primer binds at 776bp downstream of G27 cagA (located in glr orf)
cagAdupR1[r]	GGG GGT TGT ATG ATA TTT TCC	Reverse primer anealing in G27 cagA orf 426-446bp
G27dF2[s]	AGG ATA TTA CCG CTT GGC GG	Forward primer which anneals at G27 *cagA* orf 3570-3589bp
dR2[s,u]	GGT TGT TGG TTA ATG GTT TCG	Reverse primer annealing in G27 *cagA* orf 9-29bp
G27R2[s]	CCT ACA ACA CCC AAA CCA C	Reverse primer which anneals at G27 cagA orf 2798-2816bp
F2[s]	CAG CAA GGT AAC GCA AGC A	Forward primer which anneals at G27 *cagA* orf 2271-2289bp
dF2[u]	AGG ATA TTA CTG CTT GGC GG	PMSS1 *cagA* orf 3,459-3,478.
cagAupF2[u]	AGA GCC AGA ACC CAA ACA CAT	1,227 bp upstream of PMSS1 *cagA* (outside the repeating unit)
dapB-F[v]	GGA AGC ACA CGA TAG GGT	Forward primer which anneals at G27 *dapB* orf 608-625bp
glnA-R[v]	CAA CGG ATA CGA TGA TGG	Reverse primer which anneals at G27 *glnA* orf 584-601bp
cagD-R[v]	GTT GTT TGT TGG TCG TGC TGA TGC	Reverse primer which anneals at G27 *cagD* orf 422-445bp

p. Used in the generation of constructs A, B, C and D

q. Used in the screening the sucessful transformants of G27 isogenic mutants

r. Used in sequencing of *cagA* upstream region in G27 isogenic mutants

s. Used in colony PCR to screen single colony derivatives of G27/revertant, G27/2CHA-ud and G27/2CHA-d

t. Used in sequencing of duplication and deletion amplicons belong to G27/2CHA-ud and G27/2CHA-d *cagA*

u. Used in colony PCR to screen single colony derivatives of PMSS1/*cagA* -S^F-1 and -S^L-2 mutant strains

v. Used in *cag* PAI typing
